# *Escherichia coli* mediated resistance of *Entamoeba histolytica* to oxidative stress is triggered by oxaloacetate

**DOI:** 10.1371/journal.ppat.1007295

**Published:** 2018-10-11

**Authors:** Yana Shaulov, Chikako Shimokawa, Meirav Trebicz-Geffen, Shruti Nagaraja, Karen Methling, Michael Lalk, Lea Weiss-Cerem, Ayelet T. Lamm, Hajime Hisaeda, Serge Ankri

**Affiliations:** 1 Department of Molecular Microbiology, Ruth and Bruce Rappaport Faculty of Medicine, Technion, Haifa Israel; 2 Department of Parasitology, Graduate School of Medicine, Gunma University, Showa-machi, Maebashi, Gunma, Japan; 3 University of Greifswald, Institute of Biochemistry, Greifswald, Germany; 4 Faculty of Biology, Technion- Israel Institute of Technology, Technion City, Haifa, Israel; 5 Department of Parasitology, National Institute of Infectious Diseases, Toyama, Shinjuku, Tokyo, Japan; University of Virginia, UNITED STATES

## Abstract

Amebiasis, a global intestinal parasitic disease, is due to *Entamoeba histolytica*. This parasite, which feeds on bacteria in the large intestine of its human host, can trigger a strong inflammatory response upon invasion of the colonic mucosa. Whereas information about the mechanisms which are used by the parasite to cope with oxidative and nitrosative stresses during infection is available, knowledge about the contribution of bacteria to these mechanisms is lacking. In a recent study, we demonstrated that enteropathogenic *Escherichia coli* O55 protects *E*. *histolytica* against oxidative stress. Resin-assisted capture (RAC) of oxidized (OX) proteins coupled to mass spectrometry (OX-RAC) was used to investigate the oxidation status of cysteine residues in proteins present in *E*. *histolytica* trophozoites incubated with live or heat-killed *E*. *coli* O55 and then exposed to H_2_O_2_-mediated oxidative stress. We found that the redox proteome of *E*. *histolytica* exposed to heat-killed *E*. *coli* O55 is enriched with proteins involved in redox homeostasis, lipid metabolism, small molecule metabolism, carbohydrate derivative metabolism, and organonitrogen compound biosynthesis. In contrast, we found that proteins associated with redox homeostasis were the only OX-proteins that were enriched in *E*. *histolytica* trophozoites which were incubated with live *E*. *coli* O55. These data indicate that *E*. *coli* has a profound impact on the redox proteome of *E*. *histolytica*. Unexpectedly, some *E*. *coli* proteins were also co-identified with *E*. *histolytica* proteins by OX-RAC. We demonstrated that one of these proteins, *E*. *coli* malate dehydrogenase (EcMDH) and its product, oxaloacetate, are key elements of *E*. *coli*-mediated resistance of *E*. *histolytica* to oxidative stress and that oxaloacetate helps the parasite survive in the large intestine. We also provide evidence that the protective effect of oxaloacetate against oxidative stress extends to *Caenorhabditis elegans*.

## Author summary

*Entamoeba histolytica* is a unicellular parasite which infects millions of humans worldwide via contaminated food and water. It resides in the colon and most infected individuals are asymptomatic. In some people, the parasite can spread into both intestinal and extraintestinal tissues, and results in amebiasis. *E*. *histolytica* feeds on bacteria in the colonic microflora. Since changes in the composition of the colonic microflora coincide with the onset of symptomatic amebiasis in affected individuals, a potential role of this microflora in disease manifestation has been suggested. Some modulating effects of the intestinal bacteria and the nature of the native intestinal flora on amebic virulence have been reported, but the exact mechanisms have not been described. Here, we report that *E*. *coli* confers increased resistance against oxidative stress to the parasite via the production of oxaloacetate. This antioxidant metabolite is the result of the oxidation of malate by malate dehydrogenase. Our results indicate that *E*. *histolytica* can use oxaloacetate of bacterial origin to increase its resistance against oxidative stress and that this oxaloacetate promotes its survival in the large intestine of mice with experimentally-induced amebiasis. We also present evidence that oxaloacetate can protect *Caenorhabditis elegans*, another bacteria-feeding organism, against oxidative stress.

## Introduction

*Entamoeba histolytica* is a protozoan parasite, which inhabits the gastrointestinal tract, and an *E*. *histolytica* infection is a substantial health risk in almost all countries where the barrier between food and water and human feces is inadequate. The major clinical manifestations of an *E*. *histolytica* infection are amebic colitis, amebic liver abscess, and extraintestinal amebiasis. It is estimated that amebiasis accounted for 55500 deaths and 2.237 million disability-adjusted life years (the sum of years of life lost and years lived with disability) in 2010[1]. This mortality rate makes an *E*. *histolytica* infection the second leading cause of death due to a parasitic infection. *E*. *histolytica* is a dimorphic organism whose life cycle has two stages: a trophozoite, a cell-invasive form which can be found in the human intestine, and a cyst, an infective form which is found in the external environment. The conversion between the two stages is usually reversible[2]. Infection of the host occurs upon ingestion of water or food contaminated with cysts. After ingestion, the cysts pass through the stomach, excyst in the small intestine where they produce ameboid trophozoites, which then colonize the large intestine. In the colon, the trophozoites can either asymptomatically colonize the gut, re-encyst, and be expelled in the feces or cause invasive disease[3]. Although the exact conditions, which trigger the onset of invasive disease, are still unknown, the interaction between the parasite’s virulence factors and the host’s response contribute to the development of disease[4].

The human gastrointestinal tract is a nutrient-rich environment which harbors a complex and dynamic population of 38 trillion microbes [5]. About 500–1000 bacterial species colonize the adult intestine, with 30–40 species comprising up to 97% of the total population[6]^,^[7]. The majority reside in the colon where densities approach 10^11^ - 10^12^ cells/ml[8]. Following colonization of the gut, the parasite is constantly interacting with the gut microbiota whose contribution to the manifestation of disease is poorly understood. The trophozoites are quite selective in their interactions with different bacterial species and only those bacteria which have the appropriate recognition molecules get attached to the trophozoites and are ingested[9]. The relationship between *E*. *histolytica* and the gut microbiota was the subject of many studies which concluded that the gut microbiota affects greatly several aspects of *E*. *histolytica*’s physiology[10, 11]^,^[12, 13]^,^[14]. In areas where amebiasis is endemic, mixed intestinal infections of *E*.*histolytica* and enteropathogenic *Escherichia coli* (EPEC) are common[15]. Bacteria in the intestinal flora including EPEC have been proposed as inducers of amebic virulence, but the causes or mechanisms which are responsible for the induction are still undetermined [16, 17]. The presence of enteropathogenic bacteria[15] or the presence of *Prevotella copri*[18], a normal component of the gut microbiota, has been correlated to a symptomatic outcome of *E*. *histolytica* infection in young children. In contrast, mice which were inoculated with commensal *Clostridia* spp. and segmented filamentous bacteria are protected against an *E*. *histolytica* infection[19]. Amebiasis is marked by acute inflammation with the release of cytokines (tumor necrosis factor alpha (TNFα), interleukin 8 (IL-8), IL-1β, interferon gamma (IFN-γ), reactive oxygen species (ROS), and nitric oxide (NO) from activated cells of the immune system. Depending on their concentration, ROS and NO have been reported to (a) trigger stress responses, (b) control the activity of *E*. *histolytica* virulence factors, and (c) be cytotoxic [20]^,^[21–24]. Recent evidence suggests that the gut microbiome can control oxidative stress and inflammation in the gut [25–29]. Recently, we demonstrated that enteropathogenic *Escherichia coli* O55 protects *E*. *histolytica* against oxidative stress and that this bacterium exerts a strong influence on the transcriptome of oxidatively stressed parasites [30]. In this report, we inform on the mechanism of *E*. *coli*-mediated resistance of *E*. *histolytica* to oxidative stress by examining the redox proteome of the parasite exposed to *E*. *coli* and oxidative stress. We found that live *E*. *coli* trigger significant changes in the redox proteome profile of oxidatively stressed *E*. *histolytica*. We also found that *E*. *coli* malate dehydrogenase (MDH) and its product, oxaloacetate, are essential for protecting the parasite against oxidative stress.

## Results

### OX-RAC analysis of OX-proteins in *E*. *histolytica* trophozoites preincubated with *E*. *coli* O55 and then exposed to oxidative stress

**Fig 1:**
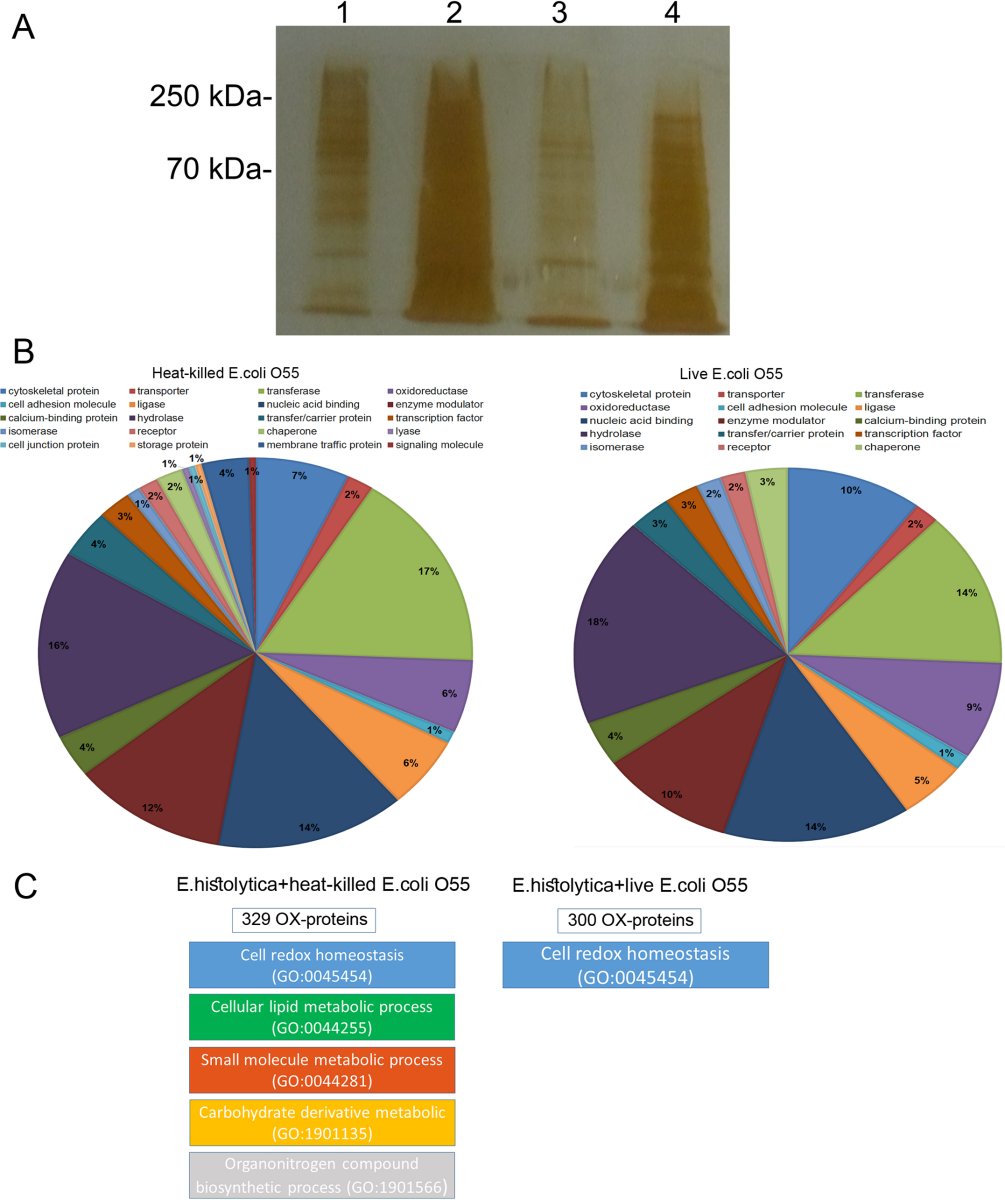
Analysis of oxidized (OX)-proteins in *E*. *histolytica* after RAC by SDS-PAGE and silver staining. **A.** Silver staining of oxidized (OX)-proteins. *E*. *histolytica* trophozoites strain HM-1:IMSS were preincubated with live or heat-killed *E*. *coli* O55 for 30 minutes and later exposed to H_2_O_2_ (2.5 mM H_2_O_2_) for 60 minutes. OX-proteins in the cell lysates were subjected to resin-assisted capture (RAC) in the presence of 10 mM DTT (+DTT) or the absence of DTT (–DTT). 1. *E*. *histolytica* + heat- killed *E*. *coli* O55 + H_2_O_2_ (2.5 mM H_2_O_2_) (-DTT); 2. *E*. *histolytica* + heat-killed *E*. *coli* O55 + H_2_O_2_ (2.5 mM H_2_O_2_) (+DTT); 3 *E*. *histolytica* + live *E*. *coli* O55 + H_2_O_2_ (2.5 mM H_2_O_2_) (-DTT); 4. *E*. *histolytica* + live *E*. *coli* O55 + H_2_O_2_ (2.5 mM H_2_O_2_) (+DTT); **B**. Functional categories of OX- proteins in *E*. *histolytica* trophozoites incubated with heat-killed *E*. *coli* and in *E*. *histolytica* trophozoites incubated with live *E*. *coli*. OX-proteins in *E*. *histolytica* were classified according to the protein class they encode using the PANTHER sequence classification tool and their biological role. **C**. Results of a PANTHER statistical overrepresentation test which compared OX-proteins in *E*. *histolytica* trophozoites preincubated with heat-killed *E*. *coli* and OX proteins in *E*. *histolytica* trophozoites preincubated with live *E*. *coli*.

The results of our recent investigation indicate that preincubation of *E*. *histolytica* trophozoites with live *E*. *coli* O55, but not with heat-killed *E*. *coli* O55, confers resistance to H_2_O_2_-induced oxidative stress to the parasite [30]. In order to obtain insights into the mechanisms of survival of oxidatively stressed *E*. *histolytica* trophozoites, we did an OX-RAC analysis of the proteins in *E*. *histolytica* trophozoites which were exposed to live or heat-killed *E*.*coli* O55 for 30 minutes and then exposed to 2.5 mM H_2_O_2_ for 60 minutes at 37°C (Fig 1A)[22]. The purification of OX-proteins by OX-RAC analysis, which has been previously described in detail [22], has three steps: (i) blocking by *N*-ethylmaleimide (NEM) of non-oxidized cysteine residues present in *E*. *histolytica* proteins; (ii) reduction of oxidized cysteine residues with dithiothreitol (DTT); and (iii) binding of the cysteine residues reduced by DTT to a thiopropyl sepharose resin. The OX-proteins are then eluted from the thiopropyl sepharose resin and identified by mass spectrometry. We identified 329 OX-proteins in those trophozoites which were exposed to heat-killed *E*. *coli* O55 and H_2_O_2_ (THK) and 300 OX-proteins in those trophozoites which were exposed to live *E*. *coli* O55 and H_2_O_2_ (TL) (S1 & S2 Tables). Using the PANTHER sequence classification tool[31], we found that a third of the OX-proteins are shared by THK and TL ((Fig 1B and S3 & S4 Tables). We also found that OX-proteins in the following biological processes were significantly enriched in THK (Fig 1C and S5 Table): redox homeostasis (GO:0045454) (exemplified by thioredoxin EHI_004490 or EHI_006700), lipid metabolism (GO:0044255) (exemplified by 3-ketoacyl-CoA synthase EHI_111000 or geranylgeranyl pyrophosphate synthase EHI_105060), small molecule metabolism (GO:0044281) (exemplified by glyceraldehyde-3-phosphate dehydrogenase EHI_008200 and threonine dehydratase EhTD1 EHI_092390), carbohydrate derivative metabolism (GO:1901135) (exemplified by mannosyltransferase EHI_103330 and glucosidase 2 subunit beta EHI_135420), and organonitrogen compound biosynthesis (GO:1901566) (exemplified by alpha-1,3-mannosyltransferase ALG2, EHI_162230 and dolichyl-diphosphooligosaccharide—protein glycosyltransferase subunit 1 EHI_029540). In contrast, we found that OX-proteins in the following biological process were significantly enriched in TL: redox homeostasis (GO:0045454) (exemplified by thioredoxin EHI_004490 or EHI_006700) (Fig 1C and S5 Table). The small number of shared OX-proteins and biological processes in THK and TL suggests that exposure of the parasite to live *E*. *coli* influences the parasite’s redox proteome. We also found 70 *E*. *coli* proteins that were co-purified with *E*. *histolytica* OX-proteins (S6 Table). It has been previously reported that a catalase-deficient strain of *Salmonella dysenteriae*, which cannot decompose H_2_O_2_ to water and oxygen, cannot boost the virulence of oxidatively stressed trophozoites whereas a wild-type *S*. *dysenteriae* does [32]. Accordingly, we posited that those *E*. *coli* proteins which are involved in the bacterium’s resistance to oxidative stress also participate in the parasite’s protective mechanism against oxidative stress. Five of the 70 *E*.*coli* proteins were selected because of their participation to the resistance of *E*. *coli* to oxidative stress, namely, 60 kDa chaperonin (it protects proteins which are denatured by oxidative stress from misfolding and aggregating [33, 34]); superoxide dismutase (it catalyzes the decomposition of the superoxide free radical [35]); MDH (it catalyzes the formation of oxaloacetate from malate and it protects *E*. *coli* against oxidative stress [36]); aspartate ammonia-lyase (it catalyzes the formation of ammonia and promotes the formation of antioxidant polyamines in *E*.*coli* [37] [38]), and alkyl hydroperoxide reductase subunit C (it catalyzes the NADH-dependent reduction of H_2_O_2_ to H_2_O [39]). We then investigated the ability of *E*.*coli* K12 and the following *E*. *coli* mutants: 60 kDa chaperonin (JW 4103- Δ gro), superoxide dismutase (JW1648- Δsod), MDH (JW 3205- Δ mdh), aspartate ammonia-lyase (JW4099- ΔaspA), and alkyl hydroperoxide reductase subunit C (JW0598- Δ ahpc) to protect *E*. *histolytica* against H_2_O_2_-induced oxidative stress. We found that the levels of protection that are conferred by *E*. *coli* O55 [30] and *E*. *coli* K12 on oxidatively stressed *E*. *histolytica* trophozoites are similar (Table 1). Among the *E*. *coli* K12 mutants, we found that the MDH mutant *E*. *coli* JW 3205 was the only one that did not protect *E*. *histolytica* against H_2_O_2_-induced oxidative stress (Table 1).

**Table 1:** LD_50_ of H_2_O_2_ for *E*. *histolytica* trophozoites exposed to malate, oxaloacetate and the different *E*.*coli* strains.

Condition	LD_50_ (mM)
*E*. *histolytica* trophozoites (Control)	3.5±0.36
*E*. *histolytica* trophozoites + *E*. *coli* K12	19.5±0.3*
*E*. *histolytica* trophozoites + *E*. *coli* JW 4103- Δ gro	16.7±2.7*
*E*. *histolytica* trophozoites + *E*. *coli* JW1648- Δsod	16.5±2.5*
*E*. *histolytica* trophozoites + *E*. *coli* JW4099- ΔaspA	12.7±1.6*
*E*. *histolytica* trophozoites + *E*. *coli* JW0598- Δ ahpc	17.9±1.3*
*E*. *histolytica* trophozoites + *E*. *coli* JW 3205- Δ mdh	4.7±1
*E*. *histolytica* trophozoites + oxaloacetate (0.25 mM)	11.3±2.8*
*E*. *histolytica* trophozoites + malate (0.5 mM)	3.4±0.2
*E*. *histolytica* trophozoites + *E*. *coli* JW 3205- Δ mdh complemented with wt *E*. *coli* mdh	14.8±2.4*

Data are displayed as the mean ± standard deviation of three independent experiments that were repeated twice.

* P ≤ 0.05 and is the significance of the difference from the LD _50_ of the control group according to the results of an unpaired t-test.

### *E*. *coli* MDH is essential for protecting *E*. *histolytica* against H_2_O_2_-induced oxidative stress

In order to demonstrate whether MDH is essential for protecting *E*. *histolytica* against H_2_O_2_-induced oxidative stress, *E*. *coli* JW 3205 was complemented with a plasmid which harbored wild-type *E*. *coli mdh*. MDH activity was determined in the whole lysate of *E*. *coli* K12, *E*. *coli* JW 3205, and *E*. *coli* JW 3205 complemented with *mdh* (Table 2). We found MDH activity in *E*. *coli* K12 and in *E*. *coli* JW 3205 complemented with *mdh* but not in *E*. *coli* JW 3205 (Table 2). We also found that *E*. *coli* JW 3205 complemented with *mdh*, but not *E*. *coli* JW 3205, protects *E*. *histolytica* against H_2_O_2_-induced oxidative stress (Table 1). MDH catalyzes the reversible transformation of malate into oxaloacetate and it has been reported that oxaloacetate in millimolar concentrations protects *E*. *coli* against H_2_O_2_-induced oxidative stress[36]. In order to test the hypothesis that oxaloacetate is essential for protecting *E*. *histolytica* against H_2_O_2_-induced oxidative stress, we determined the viability of *E*. *histolytica* trophozoites first exposed to different concentrations of oxaloacetate (0–2 mM for 15 minutes) and then to H_2_O_2_ (2.5 mM for one hour). We found that oxaloacetate at concentrations higher than 0.25 mM protects the parasite against H_2_O_2_-induced oxidative stress (Table 1 and S1 Fig). We also found that malate (0.5 mM) has no effect on the resistance of the parasite to H_2_O_2_-induced oxidative stress (Table 1). Paraquat has been previously used to trigger oxidative stress in *E*.*histolytica*[40]. In order to demonstrate whether oxaloacetate protects the parasite against paraquat, trophozoites were incubated with oxaloacetate (2 mM) and then exposed to paraquat (2.5 mM). We found that oxaloacetate does not protect the parasite against paraquat (S2 Fig).

**Table 2:** MDH activity in the different biological samples.

Source	Specific activity (U*)
***E*. *histolytica* trophozoites (total lysate)**	4.8
**pcontrol *E*. *histolytica* trophozoites (total lysate)**	4.8
***E*.*histolytica* trophozoites (secreted products)^**#**^**	0
***E*. *histolytica* trophozoites overexpressing HA-tagged EcMDH (total lysate)**	55.5 ±9.5
**Recombinant His-tagged EcMDH (commercial)**	6984±38
***E*. *coli* K12**	50.8±19
***E*. *coli JW 3205- Δ mdh* (total lysate)**	0
***E*. *coli* JW 3205- Δ mdh complemented with Ecmdh (total lysate)**	23.8±2.2
***E*. *coli* O55 (total lysate)**	79.4±28.6
***E*. *coli* JW 3205- Δ mdh complemented with Ehmdh (total lysate)**	9.5±2.5

*1 U = μmol NADH formed/min/mg protein.

Data are displayed as the mean ± standard deviation of three independent experiments that were repeated twice.

^#^ S6 Fig shows a Coomassie blue staining of proteins secreted by *E*.*histolytica* trophozoites.

Next, we tested whether *E*. *coli* O55 also protects *E*. *histolytica* against nitrosative stress. For this purpose, we exposed *E*. *histolytica* trophozoites to the nitric oxide (NO) donor, S-nitrosoglutathione (350 μM for 120 minutes)[41]. We found that *E*. *histolytica* trophozoites exposed to live or heat-killed *E*. *coli* O55 are not protected against S-nitrosoglutathione-induced nitrosative stress (S3 Fig). We also found that oxaloacetate (2 mM) does not protect the parasite against S-nitrosoglutathione-induced nitrosative stress (S3 Fig).

It has been reported that *E*.*coli* can boost the virulence of *E*.*histolytica* and that this boosting is contact dependent[42]. However, it is not known whether contact between *E*. *coli* and the parasite is necessary for protecting the parasite against H_2_O_2_-induced oxidative stress. This question was addressed by physically separating the trophozoites from *E*. *coli* O55 or *E*. *coli* K12 with a polycarbonate insert (0.4 μm) prior to the exposure of the parasite to H_2_O_2_. We found that *E*. *coli* O55 and *E*. *coli* K12 inside the polycarbonate insert protect the parasite against H_2_O_2_-induced oxidative stress (S4 Fig).

**Fig 2:**
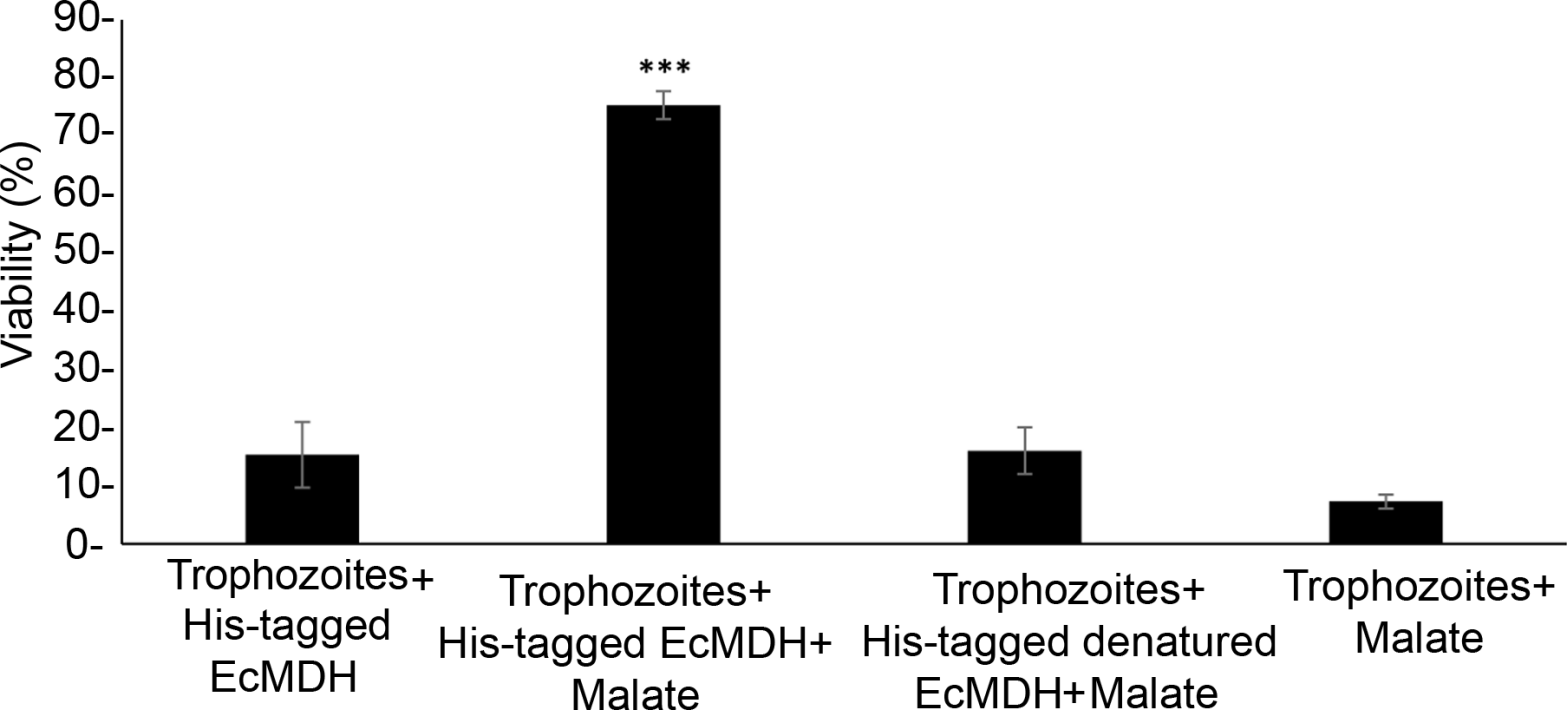
His-tagged EcMDH protects the parasite against H_2_O_2_-induced oxidative stress upon addition of malate. The viability (%) of *E*. *histolytica* trophozoites preincubated with His-tagged EcMDH (1.5 μg/ml) and malate (50 mM) was significantly higher than that of the control group (*E*. *histolytica* trophozoites incubated with the His-tagged EcMDH without malate). Data are displayed as the mean ± standard deviation of three independent experiments that were repeated twice. Unpaired t-test, ***P ≤ 0.0001.

Since *E*.*coli* MDH (EcMDH), which is secreted by *E*.*coli*[43], is essential for protecting *E*. *histolytica* against H_2_O_2_-induced oxidative stress (Table 2) and this protection is contact independent (S4 Fig), we posited that EcMDH contributes to the resistance of *E*. *histolytica* to H_2_O_2_-induced oxidative stress. This hypothesis was tested by first incubating *E*. *histolytica* with commercial His-tagged EcMDH and then exposing the parasite to H_2_O_2_-induced oxidative stress. As a prerequisite to this experiment, we checked the activity of the commercial His-tagged EcMDH and found that the recombinant protein is catalytically active (Table 2). We found that the presence of His-tagged EcMDH did not protect the parasite against H_2_O_2_-induced oxidative stress (Fig 2). However, we found that the parasite is protected against H_2_O_2_-induced oxidative stress when the parasite is incubated with both His-tagged EcMDH (1.5 μg) and L-malate (50 mM) prior to its exposure to H_2_O_2_-induced oxidative stress (Fig 2).

### Expression of *E*. *coli* MDH in *E*. *histolytica* does not protect the parasite against H_2_O_2_-induced oxidative stress

**Fig 3:**
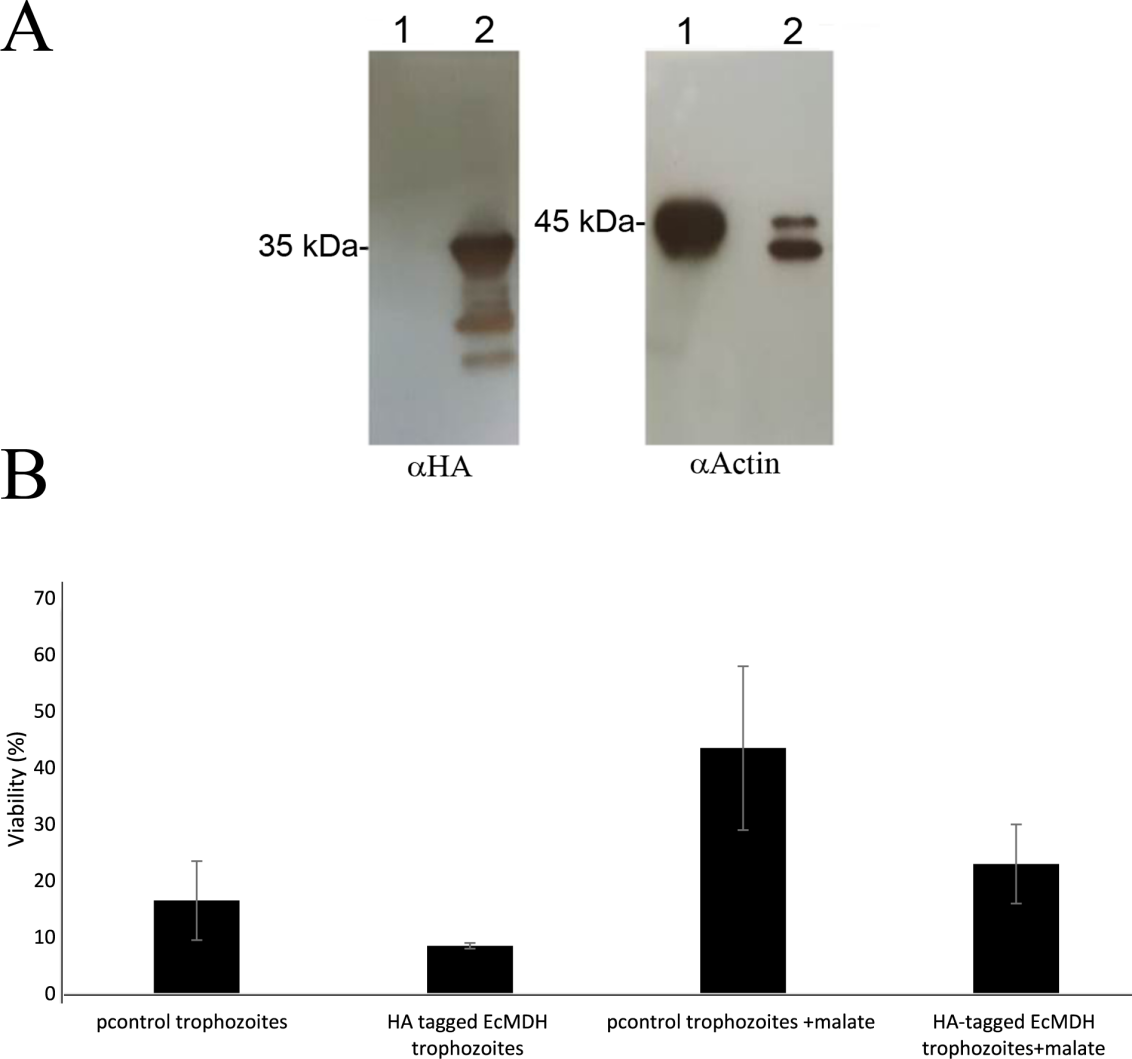
Overexpression of EcMDH does not protect *E*. *histolytica* against H_2_O_2_-induced oxidative stress. A. Western blot analysis was performed on total protein extracts that were prepared from pcontrol *E*. *histolytica* trophozoites (lane 1) and HA-tagged EcMDH trophozoites. The proteins were separated on 12% SDS-PAGE gels and analyzed by western blotting using an HA antibody or actin antibody. B. The viability of *E*. *histolytica* trophozoites was determined after their exposure to 2.5 mM H_2_O_2_ for one hour at 37°C. Some experimental groups (malate) were preincubated with 50 mM malate for 15 minutes before being exposed to H_2_O_2_. Data are displayed as the mean ± standard deviation of three independent experiments that were repeated twice.

No MDH activity was detected in a whole lysate and the secretory products of control *E*. *histolytica* trophozoites [21] (Table 2) despite the presence of six putative MDH genes in the genome of the parasite [44] (EHI_152670, EHI_067860, EHI_165350, EHI_030810, EHI_092450 and EHI_014410) and one MDH in the secretome of *E*. *histolytica* [45] (EHI_092450). When the *E*. *histolytica* MDH (EhMDH, EHI_067860) is overexpressed in *E*.*coli*, its activity is less than 40% of its *E*. *coli* homolog (Table 2). These results raise a question on whether the parasite can express an active MDH. To answer this question, *E*. *histolytica* trophozoites were complemented with HA-tagged EcMDH. The expression of HA-tagged EcMDH in the trophozoites was confirmed by western blotting (Fig 3A) and detecting MDH activity in their whole lysates (Table 2). When we compared the resistance of HA-tagged EcMDH trophozoites to H_2_O_2_-induced oxidative stress (Fig 3B) and trophozoites transformed with the pcontrol plasmid[41], we found that the sensitivity of the HA-tagged EcMDH trophozoites and the pcontrol trophozoites to H_2_O_2_-induced oxidative stress was identical. Incubation of the HA-tagged EcMDH trophozoites in presence of malate (2 mM) prior to their exposure to H_2_O_2_-induced oxidative stress did not increase their resistance to H_2_O_2_-induced oxidative stress (Fig 3B).

The intracellular concentration of oxaloacetate was determined in pcontrol trophozoites, HA-tagged EcMDH trophozoites, *E*. *coli* K12, and *E*.*coli* JW 3205. We were able to detect 12.7 nmol oxaloacetate/mg proteins in *E*. *coli* K12, but no oxaloacetate was detected in the pcontrol trophozoites, the HA-tagged EcMDH trophozoites, and *E*. *coli* JW 3205. No oxaloacetate was also detected in the supernatant of the growth medium of *E*. *coli* or *E*. *histolytica*.

### Oxaloacetate reduces the formation of OX-proteins

**Fig 4:**
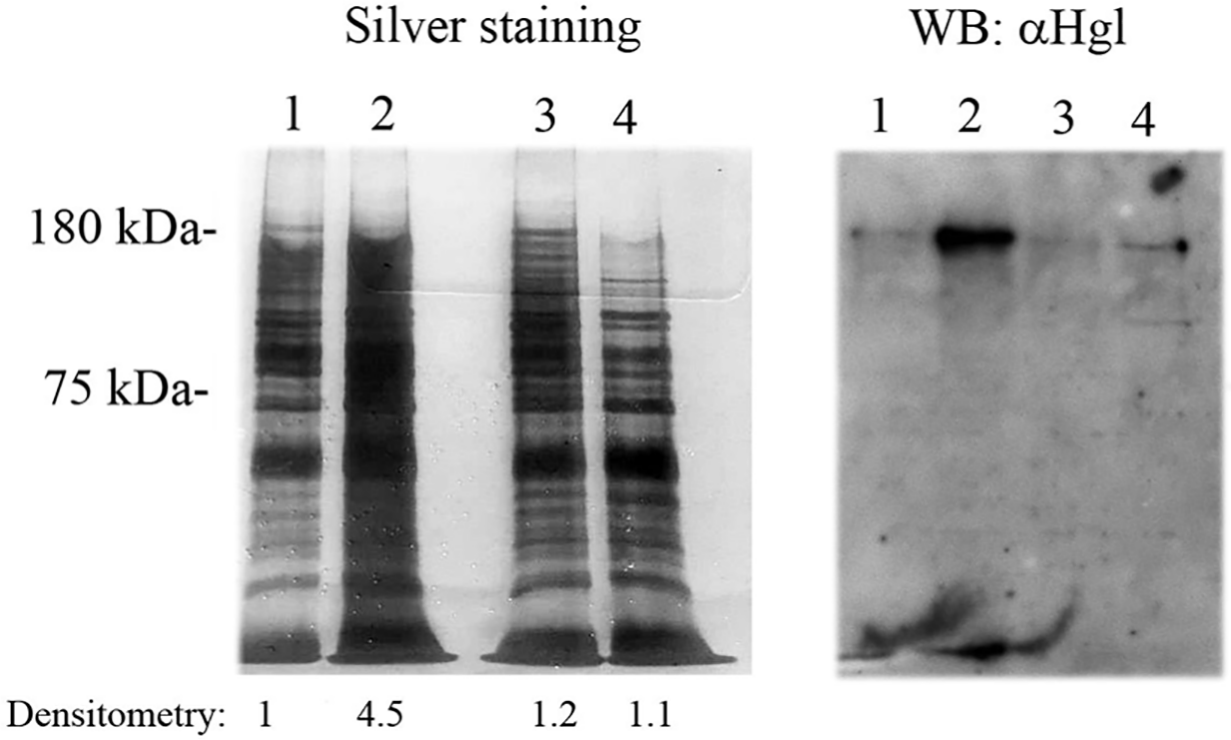
Analysis by SDS-PAGE and silver staining and by Western blot (WB) of OX-proteins in *E*. *histolytica* exposed to H_2_O_2_ or to H_2_O_2_ and oxaloacetate. *E*. *histolytica* trophozoites strain HM-1:IMSS were exposed to H_2_O_2_ (2.5 mM) or to H_2_O_2_ (2.5 mM) and oxaloacetate (2 mM) for 30 minutes. OX-proteins in the cell lysates were subjected to resin-assisted capture (RAC) in the presence of 10 mM DTT (+DTT) or the absence of DTT (–DTT). 1. *E*. *histolytica* + H_2_O_2_ (2.5 mM)(-DTT); 2. *E*. *histolytica* + H_2_O_2_ (2.5 mM)(+DTT); 3 *E*. *histolytica* + H_2_O_2_ (2.5 mM) + oxaloacetate (2 mM) (-DTT); 4. *E*. *histolytica* + H_2_O_2_ (2.5 mM) +oxaloacetate (2 mM) (+DTT). Left panel: Silver staining of OX-proteins after RAC. The intensity of the protein bands were quantified by densitometry using Image J software [89]. This image is representative of two independent experiments. Right panel: Western blot analysis was performed on OX-proteins after RAC. The proteins were separated on 8% SDS-PAGE gels and analyzed by western blotting using a polyclonal Gal/GalNAc lectin antibody. The figure displays a representative result from two independent experiments. The input control (amount of Hgl in cell lysates of trophozoites exposed to H_2_O_2_ (2.5 mM) or to H_2_O_2_ (2.5 mM) and oxaloacetate (2 mM) for 30 minutes before RAC) is shown in S7 Fig.

Ketoacids act as non-enzymatic antioxidants due to their ability to scavenge H_2_O_2_ [46, 47]. We determined the amount of H_2_O_2_ in presence or absence of oxaloacetate and confirmed that the amount of H_2_O_2_ dropped in presence of oxaloacetate (S5 Fig). The antioxidant property of oxaloacetate was also evaluated by determining the formation of OX-proteins in trophozoites exposed to H_2_O_2_ and trophozoites exposed to H_2_O_2_ and oxaloacetate using OX-RAC (Fig 4; left panel). The amount of OX-proteins in those trophozoites exposed to H_2_O_2_ was four times bigger than that in those trophozoites exposed to H_2_O_2_ and oxaloacetate. Gal/GalNac lectin is a surface protein which is essential for the binding of the parasite to target cells [48] and its binding activity is inhibited by the oxidation of the cysteine residues present in the carbohydrate recognition domain of the heavy subunit Gal/GalNAc lectin (Hgl) (EHI_012270) [22]. We decided to determine the redox status of Hgl in trophozoites exposed to H_2_O_2_ and trophozoites exposed to H_2_O_2_ and oxaloacetate by western blotting of the OX-proteins (Fig 4; right panel). We detected a strong Hgl signal in those trophozoites exposed to H_2_O_2_ whereas Hgl is barely detectable in those trophozoites exposed to H_2_O_2_ and oxaloacetate. Collectively, these results indicate that oxaloacetate reduces the formation of OX-proteins in trophozoites exposed to H_2_O_2_.

### Oxaloacetate prevents the loss of the cytopathic activity of oxidatively stressed *E*. *histolytica* trophozoites

**Fig 5:**
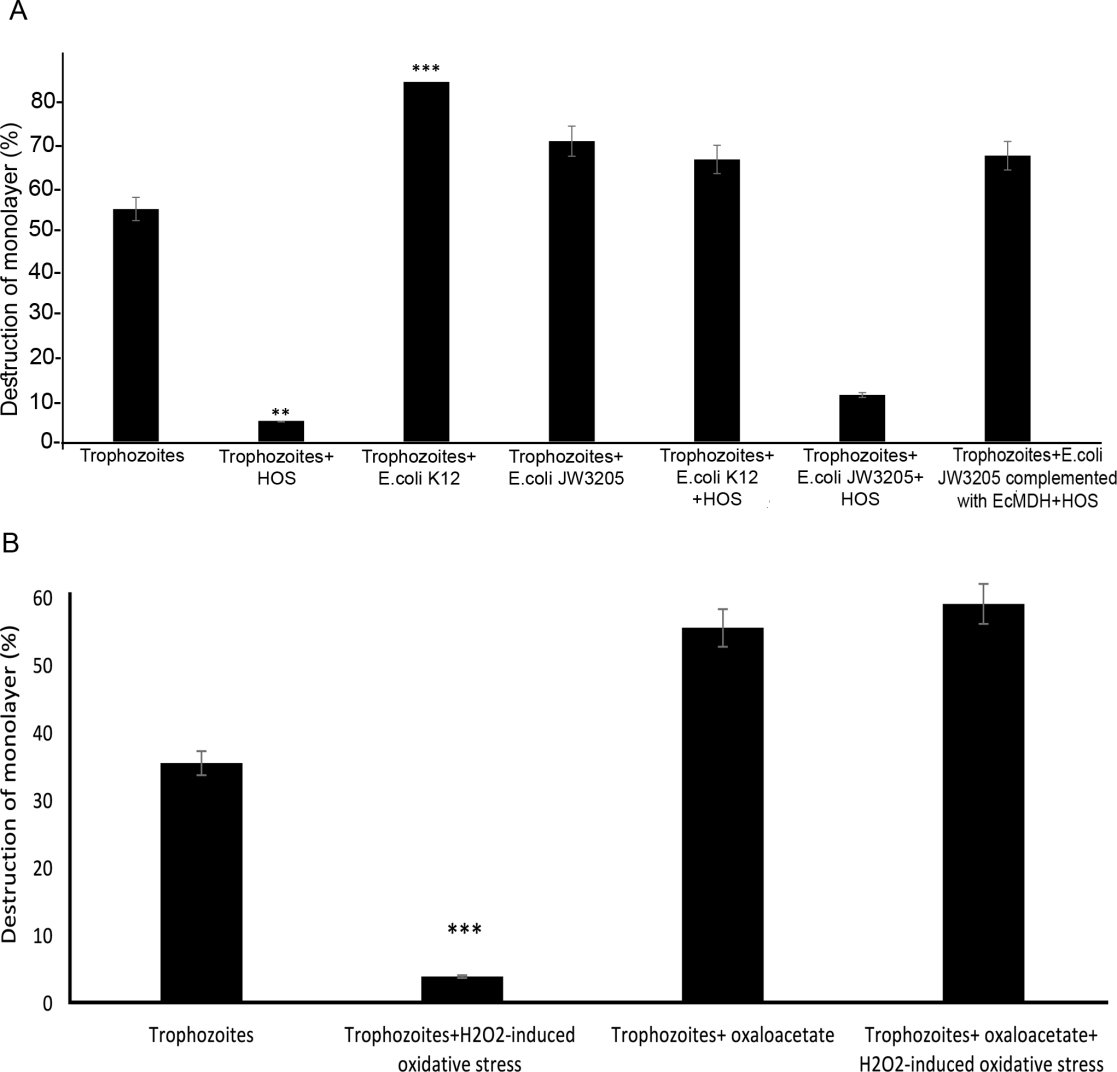
**Effect of H**_**2**_**O**_**2**_**-induced oxidative stress, *E*. *coli* and oxaloacetate on the cytopathic activity of *E*. *histolytica*: A** Cytopathic activity of *E*. *histolytica* trophozoites was measured by their ability to destroy a monolayer of HeLa cells. *E*. *histolytica* trophozoites were preincubated with *E*.*coli* O55 for 15 minutes before the addition of 2.5 mM H_2_O_2_ (HOS: H_2_O_2_-induced oxidative stress). Data are displayed as the mean ± standard deviation of three independent experiments that were repeated twice. Trophozoites versus (vs) Trophozoites + H_2_O_2_-induced oxidative stress (Unpaired t-test, **P ≤0.01), Trophozoites vs Trophozoites+ *E*.*coli* K12 (Unpaired t-test, ***P ≤0.001), Trophozoites vs Trophozoites + *E*.*coli* JW3205 + H_2_O_2_-induced oxidative stress (Unpaired t-test, **P ≤0.01) **B.** *E*. *histolytica* trophozoites were preincubated with 0.5 mM oxaloacetate for 15 minutes before the addition of H_2_O_2_. Data are displayed as the mean ± standard deviation of three independent experiments that were repeated twice. Unpaired t-test, *** P ≤0.001.

It has been previously reported that H_2_O_2_-induced oxidative stress impairs the cytopathic activity of *E*. *histolytica* [16]. We found that the cytopathic activity of *E*. *histolytica* trophozoites incubated with *E*. *coli* K12 or *E*. *coli* JW 3205 complemented with *mdh* was not impaired when they were exposed to H_2_O_2_-induced oxidative stress and was impaired when they were exposed to *E*. *coli* JW 3205 (Fig 5A). In order to test whether oxaloacetate can protect the cytopathic activity of oxidatively stressed *E*. *histolytica* trophozoites_,_ *E*. *histolytica* trophozoites were exposed or not exposed to oxaloacetate prior to their exposure to H_2_O_2_ (Fig 5B). We found that the cytopathic activity of the oxidatively stressed *E*. *histolytica* trophozoites is substantially impaired, but this impairment does not occur when the parasite was preincubated with oxaloacetate before being exposed to H_2_O_2_ (Fig 5B).

### Oxaloacetate inhibits the amebicidal activity of activated murine macrophages

**Fig 6:**
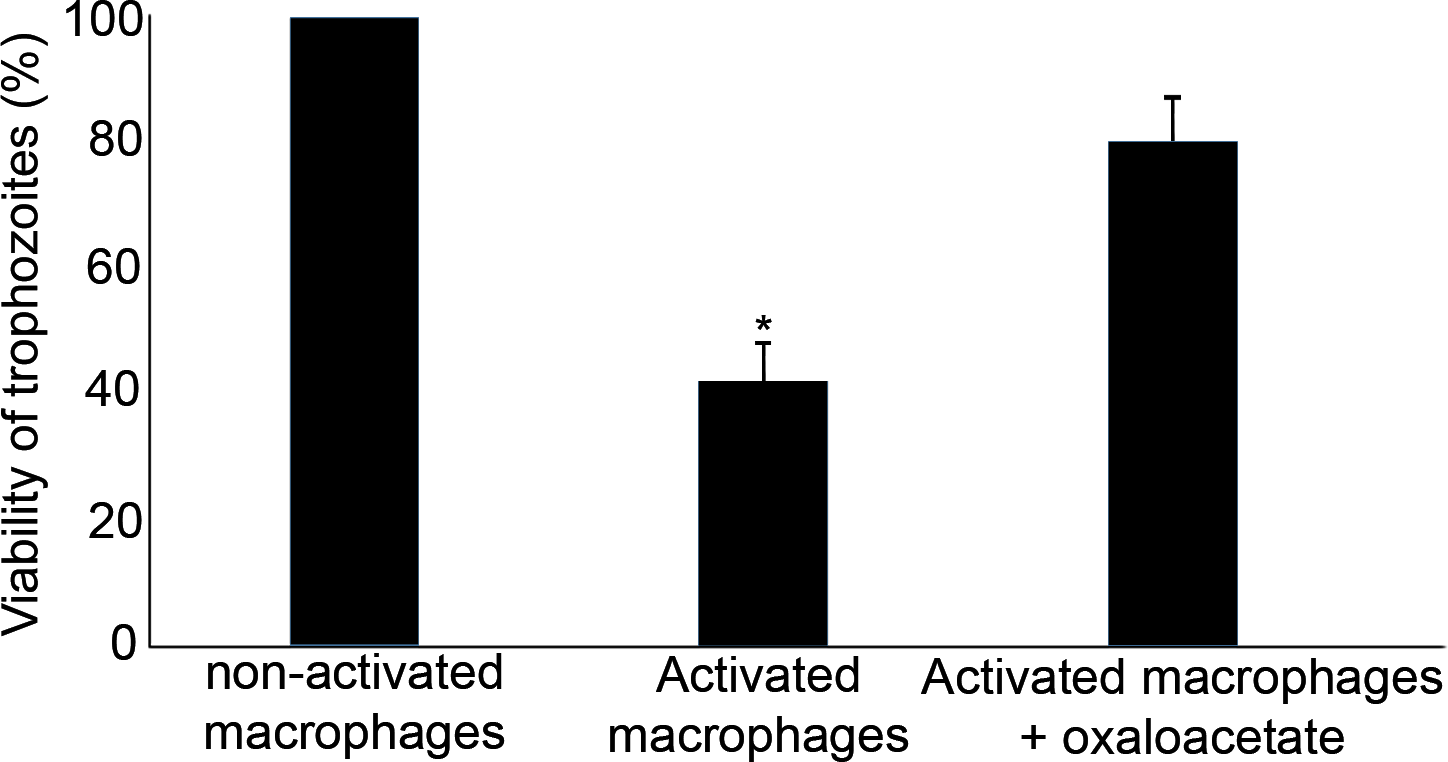
Oxaloacetate inhibits the amoebicidal activity of activated murine macrophages. Data are displayed as the mean ± standard deviation of two independent experiments that were repeated twice. Unpaired t-test, * P ≤0.05.

The amebicidal activity of murine macrophages depends on the formation of reactive oxygen and nitrogen species and the addition of catalase to the culture medium of activated murine macrophages reduces their amebicidal activity [49]. Based on these data, we determined the effect of oxaloacetate on the amebicidal activity of activated murine macrophages (Fig 6). We found that the amebicidal activity of activated murine macrophages is substantially impaired when these macrophages were incubated with oxaloacetate (Fig 6).

### Oxaloacetate helps the parasite to survive within the mouse large intestine

**Fig 7:**
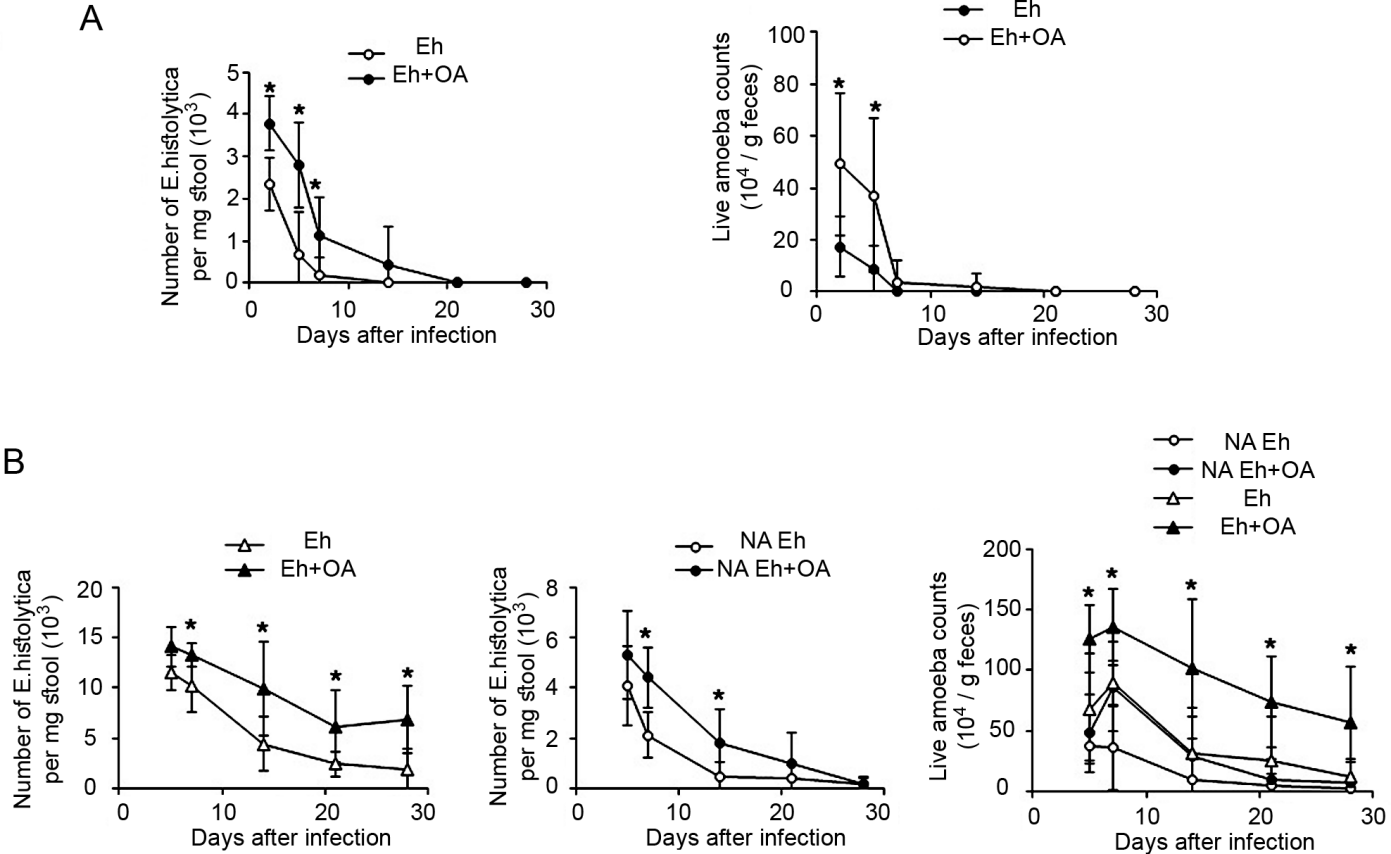
Effects of oxaloacetate on survival of *E*. *histolytica* in a mouse intestinal amebiasis model. C57BL/6 (A) and CBA/J (B) mice were intracaecally inoculated with 1 × 10^6^ trophozoites of *E*. *histolytica* (Eh) in the presence of oxaloacetate (2mM) (OA). *E*. *histolytica* without *in vivo* serial passage (NA Eh) was also used to susceptible CBA/J mice. For determining the presence of the parasite in the stool of infected mice, cultivation of stool and PCR amplification of *E*.*histolytica* 18S rRNA in DNA extracted from the stool were performed. Data are displayed as the mean ± standard deviation of six mice in one representative experiment. Asterisks indicate significant differences at *P* < 0.05 according to the results of an unpaired Student’s t-test.

The effect of oxaloacetate on the survival of *E*. *histolytica* in the large intestine was tested in a mouse strain resistant to intestinal amebiasis C57BL/6 (B6) [50] and in a mouse strain susceptible to intestinal amebiasis CBA/J [51]. The survival of the parasite was determined by counting the number of trophozoites in the stool after cultivation and by amplification of *E*.*histolytica* 18S rRNA in DNA extracted from the stool by polymerase chain reaction (PCR). We found that intracecal injection of the parasite with oxaloacetate (2 mM) helps the parasite to survive in the intestine. (Fig 7A & 7B).

### Glycerol accumulation does not occur in *E*. *histolytica* trophozoites pretreated with oxaloacetate before their exposure to H_2_O_2_-induced oxidative stress

**Fig 8:**
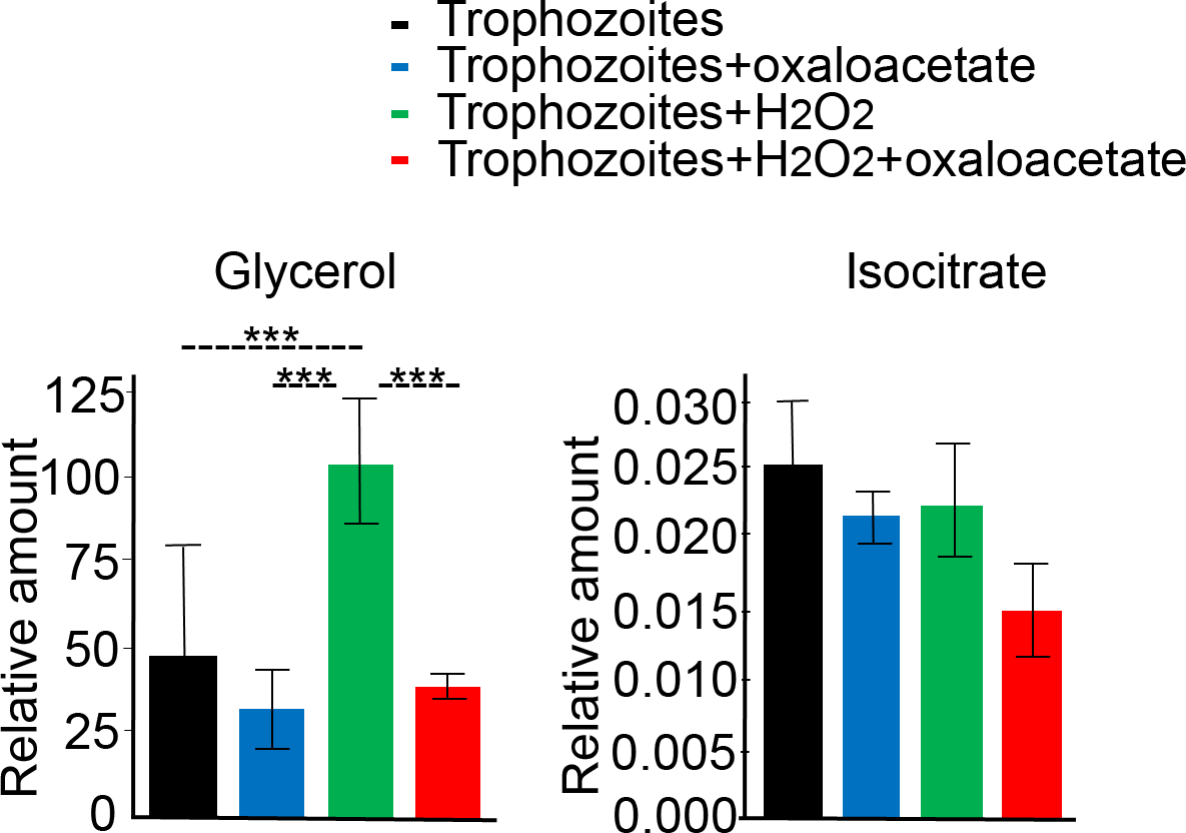
Relative amounts of glycerol and isocitrate (area metabolite/area internal standard normalized to protein content) exposed to H_2_O_2_-induced oxidative stress in the absence or presence of oxaloacetate. Trophozoites were preincubated with 2.5 mM oxaloacetate for 30 minutes without or with the addition of H_2_O_2_ (treatment with 2.5 mM for one hour). Data are displayed as the mean ± standard deviation of three independent experiments. **** P-value < 0.0001, determined by a 2-way ANOVA (Prism), corrected with Tukey’s multiple comparisons test.

It has been reported that the glycolytic activities of oxidatively stressed *E*. *histolytica* trophozoites are impaired and this impairment results in a redirection of the metabolic flux toward glycerol production[40]. Therefore, the amount of glycerol in the parasite could be used as an indicator of the level of oxidative stress sensed by the parasite. We found that the amount of glycerol in those parasites exposed to H_2_O_2_ was two-fold greater than that in the unexposed parasites (Fig 8). We also did not detect any differences in the amount of glycerol in those parasites exposed to oxaloacetate and in those trophozoites exposed to oxaloacetate and H_2_O_2_. Husain et al. [40] reported that the amount of isocitrate in control (unstressed) and oxidatively stressed trophozoites is similar. This finding suggests that the intracellular amount of isocitrate could be used as an internal standard for our metabolomic data in the control and oxidatively stressed trophozoites. We found no difference in the amounts of isocitrate in the oxidatively stressed and control parasites which confirmed the finding of Husain et al. [40] and concluded that the quality of our metabolomic data is good. (Fig 8).

### Oxaloacetate increases the survival of *C*. *elegans* exposed to H_2_O_2_

**Fig 9:**
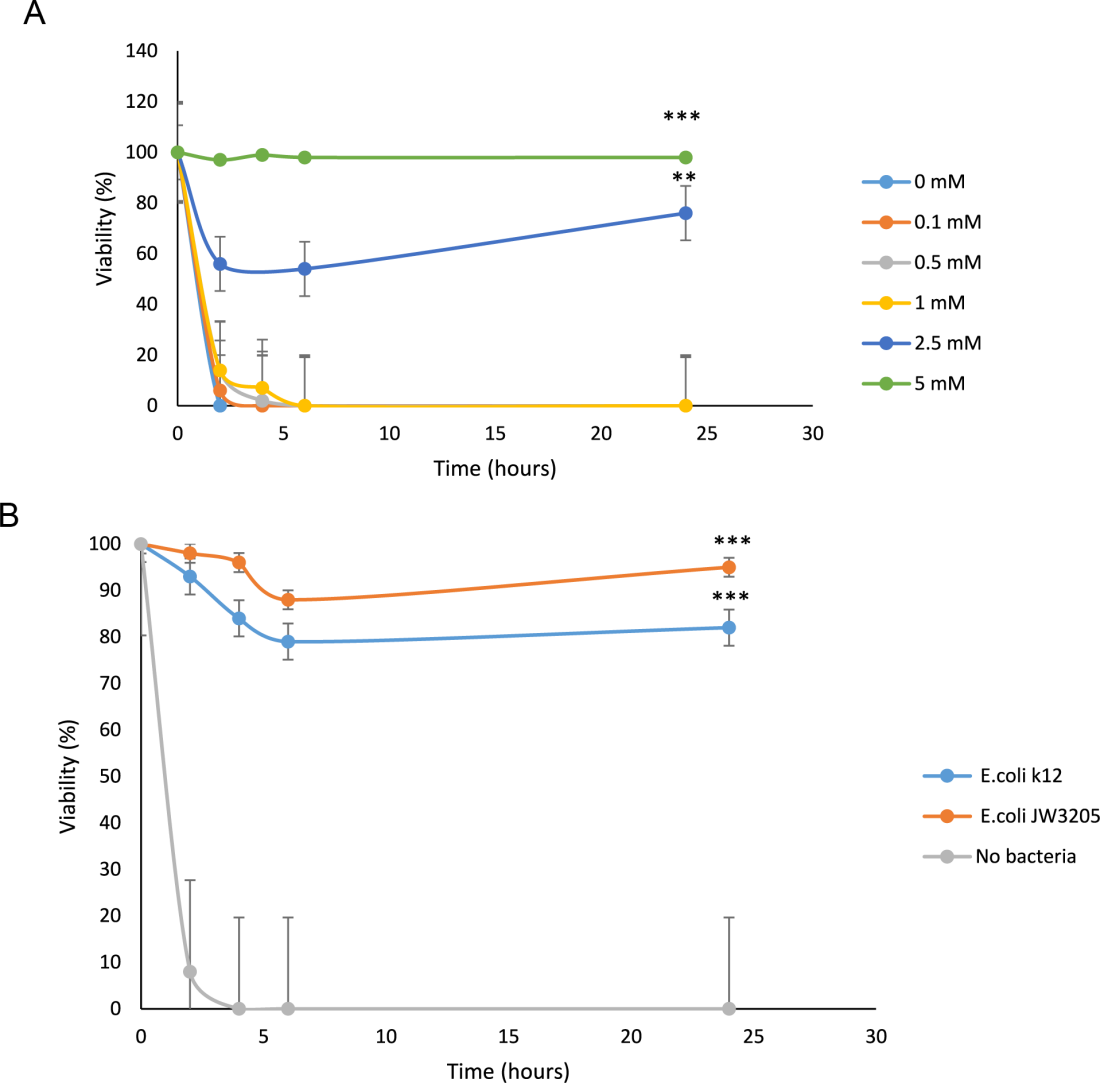
Oxaloacetate protects *C*.*elegans* against H_2_O_2_-induced oxidative stress. A. *C*. *elegans* was exposed to different concentrations of oxaloacetate in M9 medium and then to 1 mM H_2_O_2_. The curves represent the means of the survival curves from three individual experiments. Increased viability of *C*. *elegans* exposed to H_2_O_2_ can be observed when the worms were supplemented with oxaloacetate (2.5 mM and 5 mM). Control (No oxaloacetate) vs 2.5 mM oxaloacetate (Unpaired t-test, ** P ≤0.01), Control vs 5 mM oxaloacetate (Unpaired t-test, *** P ≤0.001). B. *C*. *elegans* was first exposed to *E*. *coli* K12 or *E*. *coli* JW3205 or to no bacteria in M9 medium and then to H_2_O_2_ (1 mM). The curves represent the means of the survival curves from three individual experiments. Increased viability of *C*. *elegans* exposed to H_2_O_2_ can be observed when the worms were incubated with *E*. *coli* K12 or *E*. *coli* JW3205. No bacteria (control condition) vs *E*. *coli* K12 (Unpaired t-test, *** P ≤0.001), No bacteria (control condition) vs *E*.*coli* JW3205 (Unpaired t-test, *** P ≤0.001).

The environment in which *C*. *elegans* lives contains bacteria and *C*. *elegans* uses these bacteria as its food source. Based on our findings in *E*. *histolytica*, we tested the hypothesis that bacteria can also influence the ability of *C*. *elegans* to resist H_2_O_2_-induced oxidative stress. It has been previously reported that oxaloacetate increases the life span of *C*. *elegans* [52]. Additionally, our knowledge on the effect of oxaloacetate on the resistance of the nematode to H_2_O_2_-induced oxidative stress is lacking. In order to fill these knowledge gaps, *C*. *elegans* at the L1 developmental stage were incubated with different concentrations of oxaloacetate and then exposed to H_2_O_2_. We found that the survival rate of those worms which were treated with oxaloacetate (2.5 mM or higher) is higher than those worms which were treated with oxaloacetate (1 mM or less) prior to their exposure to H_2_O_2_ (Fig 9A).

*C*. *elegans* were also incubated in M9 medium without bacteria or with *E*. *coli* K12 or *E*. *coli* JW 3205 prior to their exposure to H_2_O_2._ We found that both *E*. *coli* K12 and *E*. *coli* JW 3205 protect *C*. *elegans* against H_2_O_2_ (Fig 9B) whereas only *E*.*coli* K12 protected *E*.*histolytica* against H_2_O_2_ (Table 1). These findings suggest that the mechanism of protection of *C*. *elegans* against H_2_O_2_ is more complex than that in *E*. *histolytica* and that it does not solely depend on the activity of EcMDH.

## Discussion

In a previous report, we informed on the results of our redox proteomics analysis of oxidatively stressed *E*. *histolytica* trophozoites [22]. In order to understand the mechanisms of survival of *E*. *histolytica* trophozoites that were incubated with *E*. *coli* prior to their exposure to H_2_O_2_, we did a redox proteomics analysis of the parasite which was exposed first to heat-killed or live *E*. *coli* and then exposed to H_2_O_2_. The proteins involved in redox homeostasis, mainly, thioredoxin (TRX) and protein disulfide isomerase (PDI) were identified as OX-proteins in oxidatively stressed *E*. *histolytica* trophozoites which were exposed to heat-killed or live *E*. *coli*. This result indicates that the presence of heat-killed or live *E*. *coli* has no detectable effect on the oxidation status of these proteins. In previous investigations, we reported that TRX and PDI are oxidized and nitrosylated when *E*. *histolytica* trophozoites are exposed to oxidative and nitrosative stress [22, 53]. PDIs are oxidoreductases and isomerases which are involved in the unfolded protein response[54]. In the oxidation-reduction reaction to reduce peroxyredoxin or decompose H_2_O_2_ into H_2_O, TRX is the first substrate to be transformed by TRX reductase (TrxR)[55]. Since TRX is susceptible to oxidation[56] and is reduced as part of its antioxidant activity[57, 58], these properties may be reasons why TRX is oxidized in the oxidatively stressed parasite after its exposure to heat-killed or live *E*.*coli*.

We detected OX-proteins in *E*. *histolytica* trophozoites which were incubated with heat-killed *E*. *coli* and exposed to H_2_O_2_. Since these OX-proteins were not detected in *E*. *histolytica* trophozoites which were incubated with live *E*. *coli* and exposed to H_2_O_2_, their absence suggests that their oxidation in *E*. *histolytica* trophozoites depends on the presence of live *E*. *coli*. One of these OX-proteins is the 60S acidic ribosomal protein L9 which belongs to the “organonitrogen compound biosynthesis” class of proteins. In our previous investigation, we also identified the 60S acidic ribosomal protein L9 (EHI_193080) as an OX-protein[22]. We have previously discussed that the inhibition of protein synthesis in oxidatively stressed *E*. *histolytica* trophozoites results from the oxidation of different components of the parasite’s translational machinery which includes the 60S acidic ribosomal proteins[22]. The absence of oxidized ribosomal proteins in trophozoites which were incubated with live *E*. *coli* prior to their exposure to H_2_O_2_ suggests that the presence of live *E*. *coli* protects these proteins from oxidation. We can also deduce that this presence facilitates the survival of the oxidatively stressed parasite.

The results of previous studies have shown that a short preincubation of *E*. *histolytica* trophozoites strain HM-1:IMSS with *E*.*coli* O55 can boost the parasite’s ability to destroy monolayers of cultured cells[10]. Our findings indicate that the impaired cytopathic activity of oxidatively stressed *E*. *histolytica* trophozoites can be regained by pre-incubating the parasite with *E*. *coli* or oxaloacetate. These results and those of others[20] suggest that the parasite’s virulence and ability to resist oxidative stress are linked. This boosting of *E*. *histolytica*’s virulence by *E*.*coli* O55 is contact dependent[10] and relies on the presence of galactose lectin on the parasite’s surface. We found that the protective effect of *E*.*coli* on oxidatively stressed *E*. *histolytica* trophozoites does not rely on the binding of the bacteria to the parasite but on the formation of oxaloacetate by *E*. *coli*. Pyruvate, oxalo-ketoglutarate, and other ketoacids function as non-enzymatic antioxidants due to their ability to scavenge H_2_O_2_[46, 47]. It has also been reported that ketoacids can protect *E*. *coli*[36], several eukaryotic cell types[59]^,^[60]^,^ [61–63] and even whole organs, such as the heart and kidney[64–66] against oxidative stress. It is also interesting to note that the increase in the life span of *C*. *elegans* after exposure to oxaloacetate[52] may be due to the ability of oxaloacetate to scavenge H_2_O_2_. The ability of oxaloacetate to scavenge H_2_O_2_ is supported by our findings of a reduction in (i) the amount of OX-proteins in trophozoites exposed to H_2_O_2_ and oxaloacetate; (ii) the intracellular amount of glycerol in trophozoites exposed to H_2_O_2_ and oxaloacetate and (iii) the concentration of H_2_O_2_ is reduced in presence of oxaloacetate. The mechanism of H_2_O_2_ scavenging by oxaloacetate is not compatible with the detoxification of s-nitrosothiol groups which are formed during nitrosative stress[67]. Since it is also not compatible with the intracellular formation of superoxide by paraquat[68], this finding may explain why oxaloacetate cannot protect *E*. *histolytica* against nitrosative stress and oxidative stress induced by S-nitrosoglutathione and paraquat, respectively.

We found that oxaloacetate produced by *E*. *coli* protects *E*. *histolytica* against H_2_O_2_induced oxidative stress. It is possible that H_2_O_2_ is detoxified by oxaloacetate inside the bacteria. The need of an active EcMDH for protecting *E*. *histolytica* against H_2_O_2_ is supported by the results of our experiment in which we found that heat-killed *E*. *coli* are unable to protect the parasite against H_2_O_2_. It is also possible that H_2_O_2_ is detoxified by oxaloacetate which is secreted into the culture medium. To our knowledge, such secretion of oxaloacetate by *E*. *coli* and other microorganisms is not supported by the literature. Our attempts to detect oxaloacetate in the extracellular medium using metabolomics or an enzymatic-based kit failed because it is a very unstable metabolic intermediate[69]. However, it is possible that oxaloacetate is formed extracellularly by secreted EcMDH because (i) EcMDH is part of *E*. *coli*’s secretome [43] and (ii) the addition of recombinant EcMDH to the medium in presence of malate confers resistance to the parasite against H_2_O_2_-induced oxidative stress (this work).

Despite the presence of six putative MDH genes in the genome of the parasite [44], we and others [21] were unable to detect any MDH activity in a total lysate of the parasite. Several different explanations may account for this experimental observation: (i) EhMDHs are very sensitive to environmental conditions and are denatured once the parasite is lysed, (ii) the parasite has lost an active MDH during the course of evolution because it can relies on malate and oxaloacetate produced by the gut microbiota and (iii) EhMDHs may have a different enzymatic activity unrelated to their metabolic function as reported for a number of *E*.*histolytica* moonlighting enzymes [41, 70]. This third hypothesis is supported by the unexpected presence of two MDHs (EHI_030810 and EHI_165350) on the parasite’s surface [71].

Since *E*. *histolytica* is able to express a functional EcMDH (based on the activity measured in a total lysate of the EcMDH trophozoites), its sensitivity to H_2_O_2_-induced oxidative stress was comparable to that of pcontrol trophozoites. Since oxaloacetate was not being detected in the EcMDH-overexpressing trophozoites even when malate was present in the medium, we concluded that (i) the concentration of intracellular malate in the parasite is limiting, (ii) the parasite cannot transport external malate, and (iii) EcMDH is not functional inside the parasite. Another possibility is that the newly formed oxaloacetate is quickly converted to a non-protective metabolite in the parasite. Pyruvate:ferredoxin oxidoreductase (PFOR) is an enzyme which uses oxaloacetate as an alternative substrate to pyruvate may be responsible for this conversion[72]. Alternatively, it is possible that EcMDH is active inside the parasite, but the production of oxaloacetate is insufficient to neutralize the cytotoxic effect of H_2_O_2_. Accordingly, any neutralization or antioxidant mechanism must be operative extracellularly in order to be effective.

We found that the survival of oxaloacetate-treated parasites is better than that of parasites which were not exposed to oxaloacetate in a mouse model of amebic colitis. It has been previously shown that a strong inflammatory response occurs after injecting the parasite into the large intestine of mice [73]. This inflammatory response is essential for killing the parasite because neutrophil-depleted or dexamethasone-treated C3H or CBA mice are more susceptible than untreated mice [73]. It is possible that oxaloacetate neutralizes H_2_O_2_ which is produced by neutrophils inside the colon of the trophozoite-infected mice and consequently promotes the parasite’s survival. This hypothesis is supported by our in vitro data which demonstrated that the amebicidal activity of activated murine macrophages which depends on the formation of ROS and NO [74] is reduced in presence of oxaloacetate. The susceptibility of mice to an *E*.*histolytica* infection depends in part on the content of sialic acids in intestinal mucins and the binding of the parasite to these mucins is mediated by Hgl [75]. The better colonization of oxaloacetate-treated parasites than untreated parasites in the large intestine of mice may be explained by the presence of a functional Hgl in the oxaloacetate-treated parasite. Specifically, Hgl in the untreated parasite is inactivated or becomes non-functional when it is oxidized [22].

Although we don’t know the exact concentration of oxaloacetate in the human large intestine due to the instability of oxaloacetate, the concentration of malate, its precursor, is in the millimolar range [76]. Based on this information, it is tempting to speculate that enough oxaloacetate is produced in the human large intestine to protect the parasite against H_2_O_2_-induced oxidative stress.

To conclude, we have done the first redoxomics of *E*. *histolytica* incubated with *E*. *coli* and exposed to H_2_O_2_-induced oxidative stress. Although it is difficult to deduce from our data whether changes in the redox status of *E*. *histolytica* proteins actually occur when the parasite resides in its host, this investigation highlights that the interaction between the parasite and the gut flora is more complex than the predator-prey relationship. A complex interaction has also been recently described in *C*. *elegans*. This bacteria-feeding nematode can avoid pathogenic bacteria, such as *Pseudomonas aeruginosas*, by sensing some of their secondary metabolites [77]. Other parasitic protozoa and helminths which are also in a tight relationship with the host’s intestinal microbiota may benefit from the antioxidant properties of oxaloacetate which is produced by the gut microbiota [78]. This proposition is supported by our data about the protection of *C*. *elegans* by oxaloacetate against H_2_O_2_-induced oxidative stress. Strategies that counteract the protective effect of oxaloacetate against oxidative stress may be valuable in the treatment of amebiasis.

## Materials and methods

### *E*. *histolytica* culture

*E*.*histolytica* trophozoites HM-1:IMSS strain were grown under axenic condition at 37°C in Trypticase Yeast Extract Iron Serum (TYI-S-33) medium prepared according to a previously reported protocol[79]. The trophozoites were harvested during the logarithmic phase of growth by chilling the culture tubes at 4°C and pelleted by centrifugation at 500 g for five minutes. The pellet was washed twice with ice-cold phosphate-buffered saline.

### Bacterial strains

The bacterial strains used in this study are listed in S7 Table. *E*. *coli* was grown at 37°C in Luria-Bertani (LB) medium[80].

### *C*. *elegans* culture and synchronization

*C*. *elegans* strain Bristol N2 were grown and maintained at 16°C on nematode growth media (NGM) agar using a previously reported protocol[81]. *E*. *coli* OP50 was used as their food source. For synchronizing the worms, gravid adults were treated with a freshly prepared 20% sodium hypochlorite solution to isolate embryos. Embryos were then incubated overnight in M9 solution in a nutator at 20°C without food to allow hatching to the L1 developmental stage and to prevent further development. The number of worms was estimated using a hemocytometer.

### Cultivation of HeLa cells

HeLa cells (a kind gift from Dr. Kleinberger, Faculty of Medicine, Technion) were maintained in continuous culture using a previously described protocol [41].

### Transfection of *E*. *histolytica* trophozoites

The transfection of *E*. *histolytica* trophozoites was done using a previously described protocol[82].

### Determination of the median lethal dose (LD_50_) of H_2_O_2_

*E*. *histolytica* trophozoites (1×10^6^) in TYI-S-33 medium (without serum) were exposed to different concentrations of H_2_O_2_ (0-5mM) for one hour at 37°C. The viability of the trophozoites was determined by the eosin dye exclusion method[41].

### Viability of *E*. *histolytica* exposed to paraquat

*E*. *histolytica* trophozoites were first cultivated in standard TYI-S-33 medium in 7 ml culture tubes for 12 hours at 37°C. The culture medium was then replaced with fresh and warm culture medium and paraquat (2.5 mM final concentration) was added and the culture was continued for 12 hours. The viability of the trophozoites was determined by the eosin dye exclusion method[41].

### Viability of *C*. *elegans* exposed to H_2_O_2_

*C*. *elegans* (100 L1 larvae) after synchronization in M9 medium were placed into each well of a 24-well plate. The worms were first exposed to different concentrations of oxaloacetate (0–5 mM) for five minutes, exposed to 2.5 mM H_2_O_2_, and then incubated in an orbital shaker at 20°C for 2, 4, 6, and 24 hours. To determine their viability after each exposure time, the worms were seeded on one side of a NGM agar plate and *E*. *coli* OP50 was seeded on the opposite side. After a 1-hour incubation at room temperature, the number of viable worms was assessed by measuring their mobility[83]. At least three biological replicates were performed for each experiment.

The viability of *C*. *elegans* was also measured after preincubation of the worms with *E*.*coli* K12 or *E*.*coli* JW3205 for five minutes. Subsequently, the worms were exposed to 2.5 mM H_2_O_2_ for 2, 4, 6, and 24 hours. The viability of the worms was assessed by measuring their mobility using the previously described method.

### Viability of *E*. *histolytica* trophozoites exposed to S-nitrosoglutathione

*E*. *histolytica* trophozoites (1×10^6^) in TYI-S-33 medium (without serum) were exposed to 350μM S-nitrosoglutathione (Sigma-Aldrich, St. Louis, MO, USA) for two hours at 37°C. The viability of the trophozoites was determined by the eosin dye exclusion method[41].

### Physical separation of *E*. *histolytica* and *E*. *coli* during their exposure to H_2_O_2_

*E*. *histolytica* trophozoites (1×10^6^) in 500 μl TYI-S-33 medium (without serum) were seeded into each well of a 24-well plate (Nunclon delta surface, Thermo Scientific, Israel). A polycarbonate SPL insert (0.4 μm) (SPL Biosciences, Israel) was introduced into each well and *E*. *coli* O55 or *E*. *coli* K12 (1×10^9^ bacteria in 500 μl TYI-S-33 medium (without serum)) were introduced into the SPL insert. No bacteria were introduced in the SPL insert of the control trophozoite culture. Oxidative stress was generated in some wells by adding 2.5 mM H_2_O_2_ directly into the SPL insert. The viability of the trophozoites was determined by the eosin dye exclusion method[41].

### Measurement of the cytopathic activity of *E*. *histolytica*

The cytopathic activity of *E*. *histolytica* trophozoites was determined using a previously described protocol [84].

### Assessment of amebicidal activity by murine RAW 264.7 macrophages in presence or absence of oxaloacetate

The amebicidal activity of activated murine macrophages was determined using previously described protocol [49]. Briefly, RAW 267.7 macrophages (a kind gift from Dr. Moran Benhar, Faculty of Medicine, Technion) were activated by a 22-hour incubation with lipopolysaccharides (LPS) (1 μg/μl) and interferon γ (INF-γ) (100 U/ml) in absence of presence of oxaloacetate (2mM). Activated macrophages (2 × 10^6^/ml) and *E*. *histolytica* trophozoites (2 × 10^4^/ml) were co-incubated at 37°C for six hours. The viability of trophozoites was determined by the eosin dye exclusion method [41].

### Mice

C57BL/6 and CBA/J mice were purchased from the Jackson Laboratory (Japan). The mice were maintained under specific pathogen-free conditions.

### Parasites and intracecal inoculation in mice

Trophozoites for intracecal injections were originally derived from laboratory strain HM1:IMSS (American Type Culture Collection) that were sequentially passaged in vivo through the mouse cecum [50]. For all intracecal injections, axenic trophozoites were grown to the log phase and counted with a hemacytometer, and 1 × 10^6^ trophozoites in 200 μl TYI-S-33 medium were injected in the presence or absence of oxaloacetate (2 mM) into the proximal, middle, and apical regions of the cecum [85] of mice anesthetized with Domitor (medetomidine hydrochloride, 0.1 mg/kg) and Dormicum (midazolam, 0.1 mg/kg). At the end of the experiment, mice were sacrificed by barbiturate overdose.

### Ethics statement

We anesthetized mice with Domitor (medetomidine hydrochloride, 0.1 mg/kg) and Dormicum (midazolam, 0.1 mg/kg). Mice were sacrified by Barbiturate overdose. All experiments that involved mice were reviewed and approved by the Committee for Ethics on Animal Experiments in the Graduate School of Gunma University, and were conducted under the control of the Guidelines for Animal Experiments in the Graduate School of Medicine, Gunma University, and the Law (No. 105) and Notification (No. 6) of the Japanese Government. The protocol number 16–041 has been assigned by the Committee for Ethics on Animal Experiments in the Graduate School of Gunma University after approval of the animal experiments described in this study.

### Detection and quantification of *E*. *histolytica* in infected mice

QIAamp DNA stool kits (Qiagen, Valencia, CA) was used for DNA extraction from the mice feces according to the manufacturer’s instructions. To quantify the presence of *E*.*histolytica* trophozoites in stool, real-time quantitative PCR was performed by using using SYBR Green Supermix (Life Technologies, TA, CA, USA) in the Quant Studio 7 Flex Real-Time PCR System (Applied Biosystems®, Life Technologies, CA, USA). Primer sets specific to *E*.*histolytica* 18S rRNA were EntaF and EhR (S8 Table). To make a standard curve, DNA extracted from *E*.*histolytica* trophozoites was serially diluted from 10^5^ to 10^0^. Based on the standard curve and the stool weight, the number of trophozoite/mg stool was calculated. The presence of live trophozoites in the stool was confirmed by cultivation of stool in TYI-S-33 medium in presence of 10^3^ unit/ml penicillin G, 1 mg/ml streptomycin, and 2.5 μg/ml amphotericin B.

### Preparation of *E*. *coli* total lysate

Overnight cultures of *E*. *coli* were diluted 100-fold in 10 ml of fresh LB medium and incubated at 37°C until the OD_600_ reached 0.5. The cells were then harvested by centrifugation, washed once with phosphate-buffered saline, and finally re-suspended in 500 μl buffer which contained 0.1M Tris (pH 7.4), 2 mM EDTA, 0.2M DTT, and 0.5mM PMSF. The cells were lysed by ultrasonic disintegration using an ultrasonic disintegrator (Topas GmbH) which was operated five times for ten seconds at 50% output power at 4°C. The resultant homogenates were centrifuged at 15,000×g for 15 minutes at 4°C, and the supernatants were used for measuring the MDH activity.

### Isolation of *E*. *histolytica* trophozoites secreted products

Proteins secreted by *E*.*histolytica* trophozoites were isolated using a previously described protocol [45].

### Determination of MDH activity

Enzyme assays were performed in a 1-cm cuvette which contained 890 μl MDH assay buffer (50 mM Tris buffer and 2 mM NAD+) and 10 μl of test sample. The reaction was initiated by the addition of 100 μl L-malate (500mM) and the rate of formation of the reduced form of nicotinamide adenine dinucleotide (NADH) was monitored at 340 nm using spectrophotometer (Pharmacia Biotech Ultrospec 2000). One unit of MDH activity is defined as 1 μmol of NAD+ converted to its reduced form/min/mg protein. The protein concentration was determined by the Bradford method [86].

The commercial *E*.*coli* His-tagged MDH was purchased from Abcam (Recombinant *E*.*coli* mdh protein ab124594). The recombinant *E*.*coli* MDH protein was diluted with MDH assay buffer to a concentration of 60 μg/ml. Viability of *E*. *histolytica* trophozoites preincubated with His-tagged EcMDH (1.5 μg) with or without malate (50 mM) was performed as described above.

### DNA constructs and complementation

For the cloning of *E*. *coli* MDH, the *E*.*coli* MDH gene was amplified from *E*.*coli* genomic DNA using the *E*.*coli* MDH 5’ and *E*.*coli* MDH 3’ primers (S8 Table). The PCR product was sub-cloned using the pGEM-T easy vector system (Promega, Madison, Wisconsin, USA).

For construction of the pJST4-*E*. *coli* MDH expression vector that was used to express HA-tagged EcMDH in the parasite, a synthetic *E*. *coli* MDH gene was ordered (Synthezza, Israel). The synthetic gene was digested with the restriction enzymes KpnI and BamHI. The released MDH gene was cloned into the pJST4 vector that has been previously linearized with KpnI and BamHI restriction enzymes. The construction of the pcontrol plasmid, which was used in this investigation, has been previously described [41].

For complementation of the mutated *E*. *coli* strain JW3205 with *E*. *coli* MDH, *E*.*coli* MDH gene was amplified from *E*.*coli* genomic DNA using primers 5’ BamHI MDH and 3’ EcoRI MDH (S8 Table) and the PCR product was cloned in the pGEM-T easy vector. *E*. *coli* MDH was digested with BamHI and EcoRI and then cloned in the vector pGFP (Genbank accession No: U17997) which had been previously linearized with BamHI and EcoRI.

For complementation of the mutated *E*. *coli* strain JW3205 with *E*. *histolytica* MDH, the *E*. *histolytica* MDH gene was amplified from a PGEX-EhMDH vector using the primers EhMDH BamHI 5’and EhMDH EcoRI 3’(S8 Table). The PCR product was cloned in the pGEM-T easy vector. *Eh* MDH was digested with BamHI and EcoRI and then cloned in the pGFP vector (Genbank accession number: U17997) which had been previously linearized with BamHI and EcoRI.

### Primers used in this study

Primers used in this study are displayed in S8 Table.

### Detection of oxidized (OX) proteins by resin-assisted capture (RAC) (OX-RAC)

The detection of OXs by OX-RAC was performed using a previously described protocol[22]. Captured proteins were eluted with 30 μl elution buffer which contained 10 mM HEPES, 0.1 mM EDTA, 0.01 mM neocuproine, 0.1% SDS and 100 mM 2-mercaptoethanol for 20 minutes at room temperature. Proteins in a 10-μl aliquot of each eluent were resolved on a 12.5% SDS-PAGE gel. Each gel was then stained with silver (Pierce Silver Stain) and each gel slice was independently analyzed by mass spectrometry (MS). A protein was considered to be oxidized when its relative amount in the dithiothreitol (DTT)-treated lysates was significantly less than that in the DTT-untreated lysates (p <0.05 according to the results of a unpaired t-test).

### In gel proteolysis and mass spectrometry analysis

In gel proteolysis by trypsin and analysis by LC-MS/MS on Q Exactive plus (Thermo) and data analysis with MaxQuant 1.5.2.8 [87] and the Uniprot database as the reference were done using a previously described protocol[22]. The data was quantified by LF analysis using the same software. The identifications are filtered for proteins identified with a false discovery rate of <0.01 and at least two identified peptides in the project. The intensities are presented as raw intensities without normalization and as LFQ with normalization, both presented as log2 intensities.

### Classification of OX-proteins according to their protein class

The OX-proteins were classified according to their protein class using PANTHER software (Protein ANalysis THrough Evolutionary Relationships) Classification System (http://www.pantherdb.org/)[31].

### Detection of OX-Gal/GalNac lectin

Following the OX-RAC procedure, proteins in a 10 μl aliquot of each eluent were resolved on an 8% SDS-PAGE gel and stained with silver or transferred onto a nitrocellulose membrane (Whatman, Protran BA83). The blots were first blocked using 3% skim milk, and then probed with 1:500 rabbit polyclonal Gal/GalNAc lectin antibody (a kind gift from Dr. N. Guillen, Pasteur Institute, Paris, France) for 16 hours at 4^°^C. After incubation with the Gal/GalNac lectin antibody, the blots were incubated with 1:5000 secondary rabbit antibody for one hour at room temperature (Jackson ImmunoResearch), and then developed by enhanced chemiluminescence.

### Detection of oxaloacetate

The detection of oxaloacetate in *E*. *coli* and in *E*. *histolytica* was done using a commercial enzymatic-based kit (Abcam, Zotal, Israel).

### Ultraviolet (UV) spectrophotometric analysis of the stability of H_2_O_2_ in presence of oxaloacetate

Oxaloacetate (0.25 mM) and H_2_O_2_ (3.5 mM) in 10 mM phosphate buffer, pH 7.4, were mixed together at 25°C and the concentration of H_2_O_2_ was determined by UV spectrophotometric analysis according to a previously described protocol [47] on a UV spectrophotometer NanoDrop 2000c (Thermo Fisher Scientific, USA).

### Metabolomics

*E*. *histolytica* trophozoites were suspended in 2 ml ice cold methanol and transferred into a 4-ml tube which contained glass beads (0.10–0.11-mm diameter) (Sartorius AG). Cell disruption was performed by using a FastPrep-24 instrument (MP Biomedicals, LLC) twice for 40 seconds each at 6.0 m/s. Methanolic cell extract was transferred to 15-ml tubes after centrifugation for five minutes at 4°C. Cell debris and glass beads were washed twice with 1 ml ultrapure water as a second extraction step.

The aqueous and methanolic cell extracts were combined. An aliquot (0.4 ml) of chloroform was added to the cell extracts and the suspension was vortexed and shaken five times for ten seconds. For separation of the aqueous and organic layers, the samples were stored at -20°C for ten minutes. After centrifugation for five minutes at 4°C and 10,015*g*, the upper layer was transferred to a 50-ml tube, diluted with water, and stored at -80°C for lyophilization.

### Gas chromatography–mass spectrometry (GC-MS) analysis

Derivatization of lyophilized samples was done using a previously described protocol[88]. Analysis was performed by using an Agilent 7890B GC system with an autosampler (model G4513A), and a coupled mass selective detector (model 5977B MSD) (Agilent). The 2-μl injection volume of the sample was split 1:10 at 250°C with an inlet split flow of 10 ml/min. Helium was used as the carrier gas at a pressure of 8.8 lb/in^2^. Chromatographic separation on a 30-m HP 5-ms column (Agilent Technologies) with a 0.25-mm inner diameter and a 2.5-μm film thickness was performed at a constant gas flow of 1 ml/min. The oven program started with an initial temperature which was held at 70°C for one minute, continued at a heating rate of 1.5°C/minute up to 76°C, followed by heating at 5°C/minute up to 220°C, and 20°C/minute up to 325°C, with a hold time of eight minutes. The analytes were transferred to the mass selective detector via the transfer line at 325°C and ionized by electron impact ionization at 230°C. After a solvent delay of six minutes, mass spectra were acquired using a quadrupole temperature of 150°C and SIM acquisition mode., The selected quantifier ion for glycerol was m/z 205.1 and for isocitrate was m/z 245.1.

Data analysis was done by using MassHunter Workstation software Quantitative Analysis 8.0. The area of the quantifier ion of each metabolite was integrated and normalized to the area of the quantifier ion of one internal standard (glycerol and isocitrate were normalized to *p*-chlorophenylalanine). This ratio represents the relative metabolite amount and was normalized to the protein content of the sample.

## Supporting information

S1 Table Legend of S2 Table. (PDF)

S2 Table Redox-proteomics data. (XLSX)

S3 Table PANTHER sequence classification of OX-proteins. (XLS)

S4 Table Common OX-proteins in trophozoites incubated with heat-killed or live *E*.*coli* O55. (XLS)

S5 Table Enriched OX-proteins according to the PANTHER sequence classification tool. (XLS)

S6 Table *E*. *coli* proteins co-purified with *E*.*histolytica* OX-proteins. (XLSX)

S7 Table Bacterial strains used in this study. (DOCX)

S8 Table Primers used in this study. (DOCX)

S1 Fig Viability of *E*. *histolytica* trophozoites exposed to different concentrations of oxaloacetate prior to their exposure to 2.5 mM H_2_O_2_. Data are displayed as the mean ± standard deviation of three independent experiments that were repeated twice. (Unpaired t-test, * P ≤0.05).
(TIF)

S2 Fig Viability of *E*. *histolytica* trophozoites exposed to 0.5 mM oxaloacetate prior to their exposure to 2.5 mM paraquat. Data are displayed as the mean ± standard deviation of three independent experiments that were repeated twice. Trophozoites vs Trophozoites + Paraquat (Unpaired t-test, *** P ≤0.001), Trophozoites vs Trophozoites+Oxaloacetate+Paraquat (Unpaired t-test, *** P ≤0.001).
(TIF)

S3 Fig Viability of *E*. *histolytica* trophozoites exposed to different concentration of oxaloacetate prior to their exposure to 350 μM S-nitrosoglutathione. Data are displayed as the mean ± standard deviation of three independent experiments that were repeated twice.
(TIF)

S4 Fig The protection of *E*.*histolytica* against OS by *E*.*coli* is contact-independent. *E*.*histolytica* trophozoites and *E*.*coli* K12 or *E*.*coli* O55 were physically separated by a polycarbonate insert (0.4μm) prior to their exposure to oxidative stress. Data are displayed as the mean ± standard deviation of three independent experiments that were repeated twice. Trophozoites vs Trophozoites + H_2_O_2_ (Unpaired t-test, ** P ≤0.01).
(TIF)

S5 Fig Spectrophotometric analysis of the stability of H_2_O_2_ in presence of oxaloacetate. The absorbance of H_2_O_2_ is followed at 225 nm.
(TIF)

S6 Fig Coomassie blue staining of proteins secreted by *E*.*histolytica* trophozoites. Lane 1: Molecular weight marker (SMOBIO-PM-2600). Lane 2: Proteins secreted by *E*.*histolytica* trophozoites (20 μg).
(TIF)

S7 Fig Amount of Hgl in the total lysate of trophozoites exposed to H_2_O_2_ or to H_2_O_2_ and oxaloacetate. Proteins (40 μg) present in a lysate of trophozoites exposed to H_2_O_2_ or to H_2_O_2_ and oxaloacetate were separated on 8% SDS-PAGE gels and analyzed by western blotting using a polyclonal Gal/GalNAc lectin antibody. The figure displays a representative result from two independent experiments. Cell lysates of trophozoites exposed to H_2_O_2_ (2.5 mM) (lane 1) to H_2_O_2_ (2.5 mM) and oxaloacetate (2 mM) (lane 2) for 30 minutes (input control for the data presented in Fig 4).
(TIF)

S1 References References cited in S7 Table. (DOCX)
