# Supplementary material for: Escherichia coli mediated resistance of Entamoeba histolytica to oxidative stress is triggered by oxaloacetate
Source: PLoS Pathog. 2018 Oct 11;14(10):e1007295. doi: 10.1371/journal.ppat.1007295 (PMC6181410; doi:10.1371/journal.ppat.1007295)
Supplement: S7 Table — (DOCX) [file ppat.1007295.s007.docx]

| Strains | Relevant phenotype | Source |
| --- | --- | --- |
| *E. coli* O55: TW04062 | Wild type *E. coli* O55 | Thomas S. Whittam STEC Center  http://www.shigatox.net/new/ |
| *E. coli* K12 | Wild type *E. coli* K12 | Sima Yaron, Biotechnology and Food Engineering , TECHNION |
| JW0598-2 | Δ ahpC744::kan | Keio Collection[[90](#_ENREF_90)] |
| JW1648-1 | ΔsodB734::kan | Keio Collection[[90](#_ENREF_90)] |
| JW3205-1 | Δ mdh-761::kan | Keio Collection[[90](#_ENREF_90)] |
| JW4099-1 | ΔaspAA765::kan | Keio Collection[[90](#_ENREF_90)] |
| JW4103-1 | Δ groL768::kan | Keio Collection[[90](#_ENREF_90)] |
| *E. coli* DH5α | huA2Δ(argF-lacZ)U169 phoA glnV44 Φ80Δ (lacZ)M15 gyrA96 recA1 relA1 endA1 thi-1 hsdR17 | New England Bio Labs |
| *E. coli* BL21 | BL21(DE3)pLysS | New England Bio Labs |
| *E. coli OP50* | Uracil auxotroph | Caenorhabditis Genetics Center [[81](#_ENREF_81)] |
