# Supplementary material for: Escherichia coli mediated resistance of Entamoeba histolytica to oxidative stress is triggered by oxaloacetate
Source: PLoS Pathog. 2018 Oct 11;14(10):e1007295. doi: 10.1371/journal.ppat.1007295 (PMC6181410; doi:10.1371/journal.ppat.1007295)
Supplement: S8 Table — (DOCX) [file ppat.1007295.s008.docx]

| Primer Name | Sequence | Direction | Restriction Site |
| --- | --- | --- | --- |
| *E.coli* MDH 5’ | ATGAAAGTCGCAGTCCTCGGC | Sense |  |
| *E.coli* MDH 3’ | CTTATTAACGAACTCTTCGC | Antisense |  |
| 5’ BamHI MDH | GGATCCAATGAAAGTCGCAGTCCTCGGCG | Sense | BamHI |
| 3’ EcoRI MDH | GAATTCTTACTTATTAACGAACTCTTCGCCCAG | Antisense | EcoRI |
| EhMDH BamHI 5’ | GGATCCAATGCAACAACCAATTCCTTGGGA | Sense | BamHI |
| EhMDH EcoRI 3’ | GAATTCTCAATTAACATATTTCGTTCATCAA | Antisense | EcoRI |
| EntaF | ATGCACGAGAGCGAAAGCAT | Sense |  |
| EhR | GATCTAGAAACAATGCTTCTCT | Antisense |  |
