# Supplementary figures and images for: Escherichia coli mediated resistance of Entamoeba histolytica to oxidative stress is triggered by oxaloacetate
Source: PLoS Pathog. 2018 Oct 11;14(10):e1007295. doi: 10.1371/journal.ppat.1007295 (PMC6181410; doi:10.1371/journal.ppat.1007295)

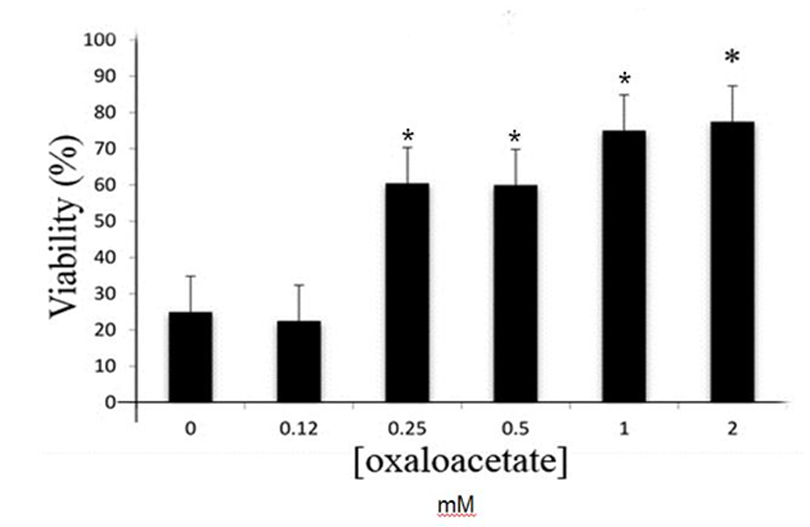

Supplement: S1 Fig — Data are displayed as the mean ± standard deviation of three independent experiments that were repeated twice. (Unpaired t-test, * P ≤0.05). (TIF) [file ppat.1007295.s009.tif]

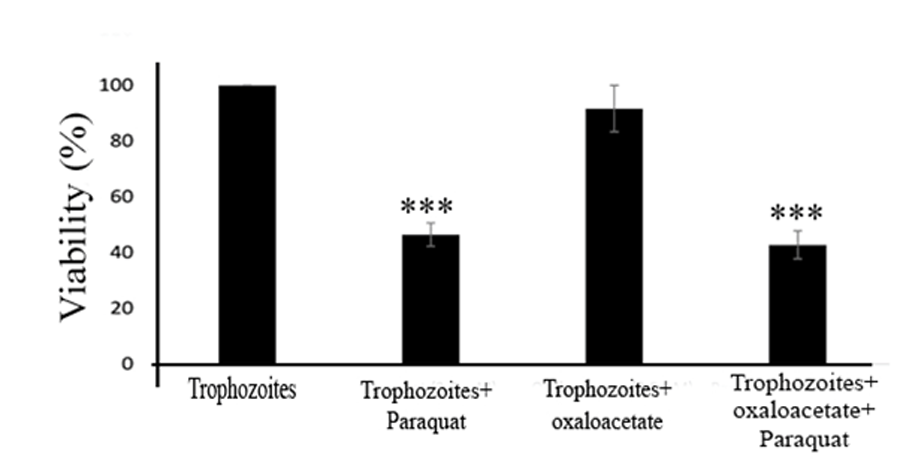

Supplement: S2 Fig — Data are displayed as the mean ± standard deviation of three independent experiments that were repeated twice. Trophozoites vs Trophozoites + Paraquat (Unpaired t-test, *** P ≤0.001), Trophozoites vs Trophozoites+Oxaloacetate+Paraquat (Unpaired t-test, *** P ≤0.001). (TIF) [file ppat.1007295.s010.tif]

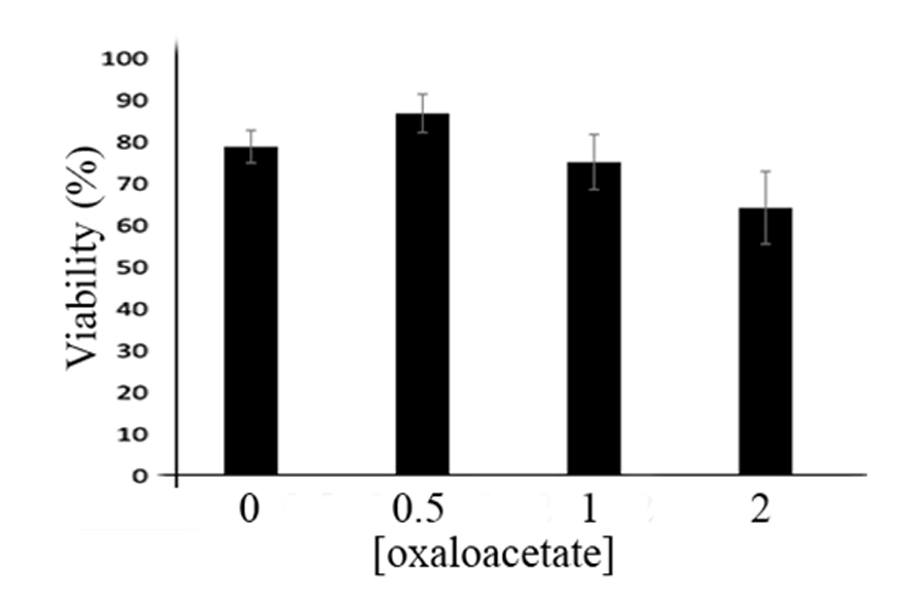

Supplement: S3 Fig — Data are displayed as the mean ± standard deviation of three independent experiments that were repeated twice. (TIF) [file ppat.1007295.s011.tif]

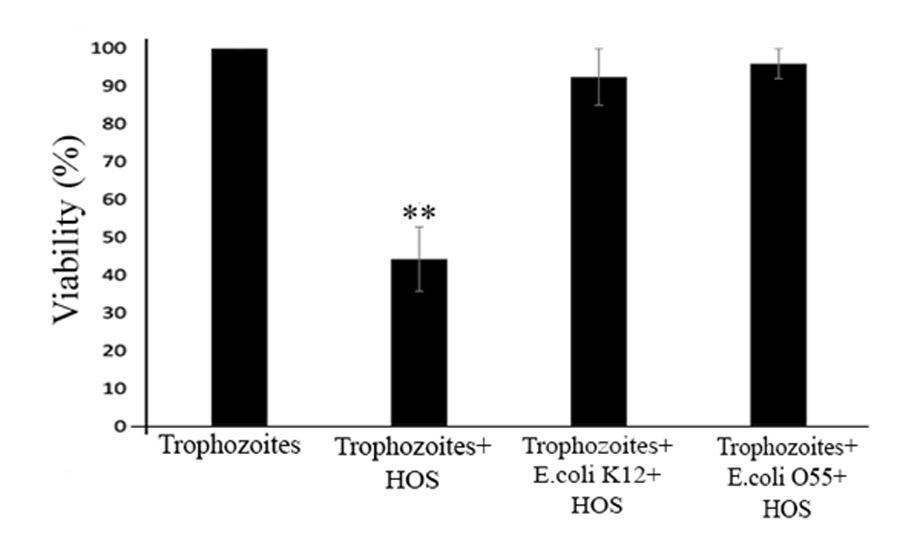

Supplement: S4 Fig — E.histolytica trophozoites and E.coli K12 or E.coli O55 were physically separated by a polycarbonate insert (0.4μm) prior to their exposure to oxidative stress. Data are displayed as the mean ± standard deviation of three independent experiments that were repeated twice. Trophozoites vs Trophozoites + H2O2 (Unpaired t-test, ** P ≤0.01). (TIF) [file ppat.1007295.s012.tif]

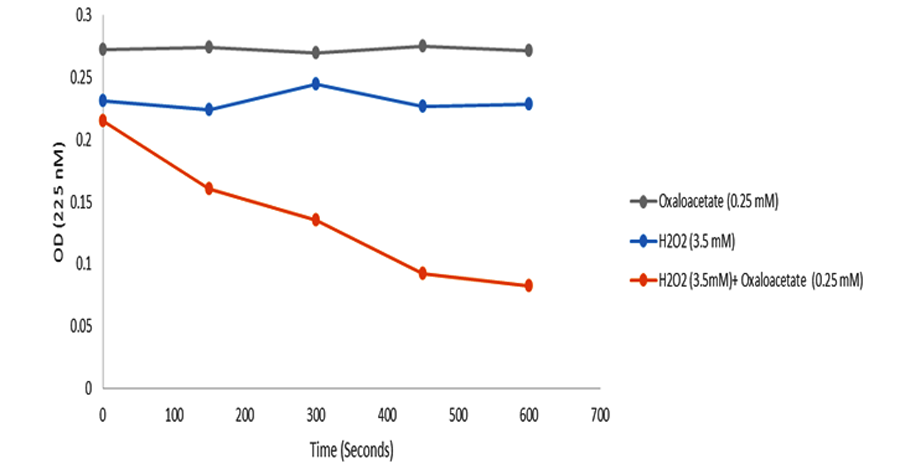

Supplement: S5 Fig — The absorbance of H2O2 is followed at 225 nm. (TIF) [file ppat.1007295.s013.tif]

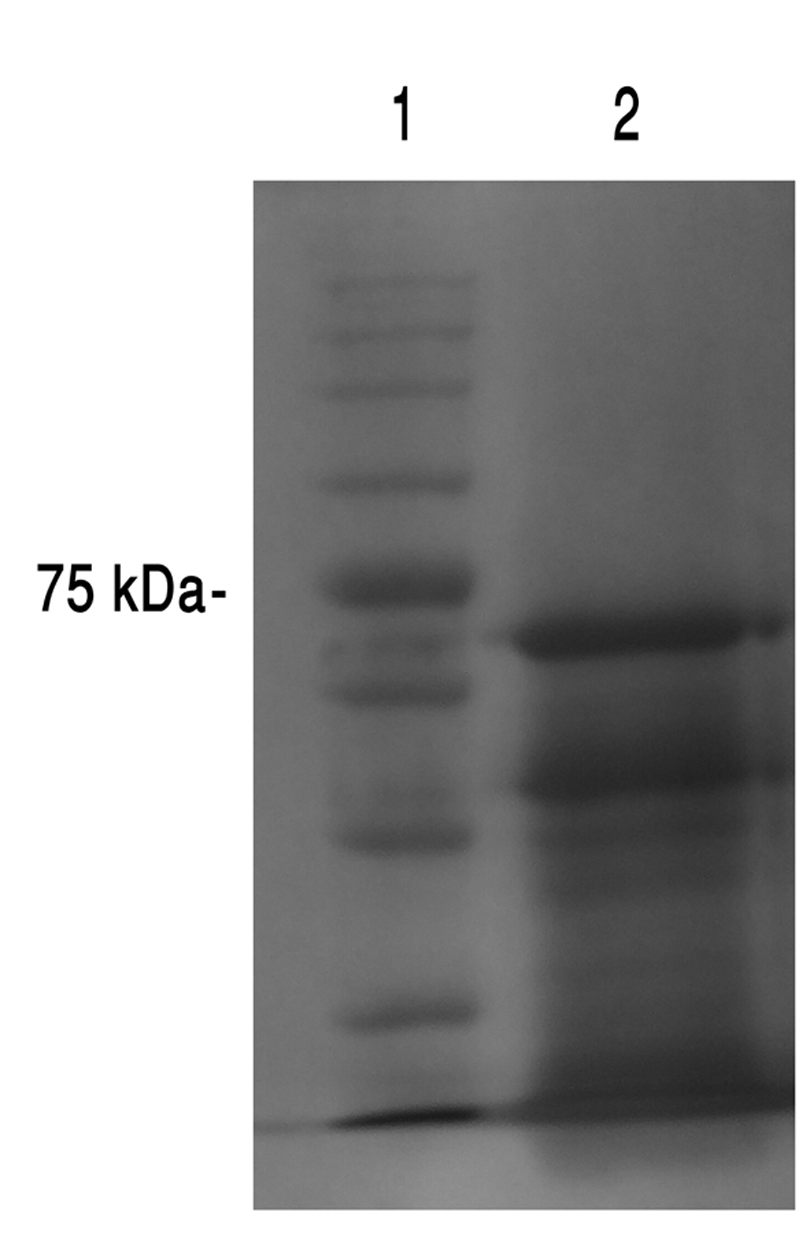

Supplement: S6 Fig — Lane 1: Molecular weight marker (SMOBIO-PM-2600). Lane 2: Proteins secreted by E.histolytica trophozoites (20 μg). (TIF) [file ppat.1007295.s014.tif]

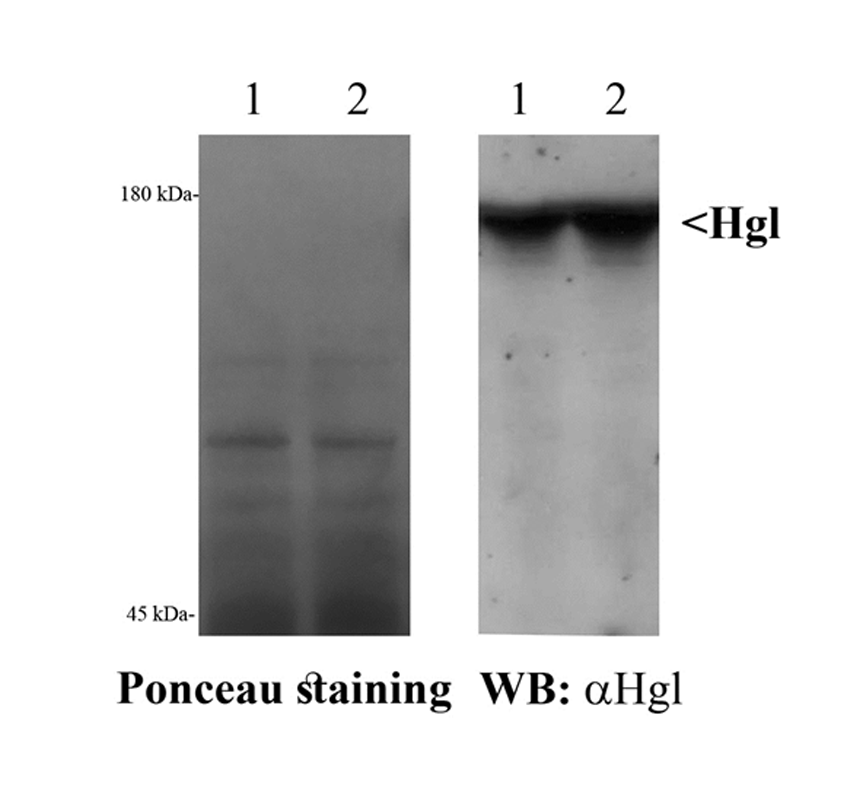

Supplement: S7 Fig — Proteins (40 μg) present in a lysate of trophozoites exposed to H2O2 or to H2O2 and oxaloacetate were separated on 8% SDS-PAGE gels and analyzed by western blotting using a polyclonal Gal/GalNAc lectin antibody. The figure displays a representative result from two independent experiments. Cell lysates of trophozoites exposed to H2O2 (2.5 mM) (lane 1) to H2O2 (2.5 mM) and oxaloacetate (2 mM) (lane 2) for 30 minutes (input control for the data presented in Fig 4). (TIF) [file ppat.1007295.s015.tif]
