# Supplementary material for: Escherichia coli mediated resistance of Entamoeba histolytica to oxidative stress is triggered by oxaloacetate
Source: PLoS Pathog. 2018 Oct 11;14(10):e1007295. doi: 10.1371/journal.ppat.1007295 (PMC6181410; doi:10.1371/journal.ppat.1007295)
Supplement: S1 References — (DOCX) [file ppat.1007295.s016.docx]

81. Brenner S. The Genetics of Caenorhabditis elegans. Genetics. 1974;77(1):71-94.

90. Baba T, Ara T, Hasegawa M, Takai Y, Okumura Y, Baba M, et al. Construction of Escherichia coli K-12 in-frame, single-gene knockout mutants: the Keio collection. Mol Syst Biol. 2006;2:2006 0008. Epub 2006/06/02. doi: msb4100050 [pii]10.1038/msb4100050. PubMed PMID: 16738554; PubMed Central PMCID: PMC1681482.
